# Downregulation of tropomyosin 2 promotes the progression of lung adenocarcinoma by regulating neutrophil infiltration through neutrophil elastase

**DOI:** 10.1038/s41419-025-07531-1

**Published:** 2025-04-08

**Authors:** Caixiu Huang, Hao Qiu, Changting Xu, Zilong Tan, Mei Jin, Jing Hu, Zhilong Huang, Yuwei Zhou, Shengyou Ge, Xiaoyuan Hu

**Affiliations:** 1 Shandong Provincial Hospital Affiliated to Shandong First Medical University, Jinan, Shandong PR China; 2 The First People’s Hospital of Changde City, Changde, PR China; 3 Nanchang University Second Affiliated Hospital, Nanchang, PR China; 4 Pingxiang People’s Hospital, Pingxiang, PR China; 5 Guangxi Medical College, Nanning, PR China

**Keywords:** Non-small-cell lung cancer, Immunoediting

## Abstract

Lung adenocarcinoma (LUAD) is a common malignant tumor in the lung that seriously endangers the health of people worldwide. The neutrophil-associated inflammatory microenvironment contributes to the activation of tumor cells. In this study, we report a role of tumor-associated neutrophils (TANs) promote tumor progression of LUAD by crosstalk between neutrophils and tumor cells. Mechanistically, in co-culture with tumor cells, downregulation of TPM2 on tumor cells increases neutrophil elastase (ELANE) levels in neutrophils regulated by p38/ MAPK signaling activation, and ELANE promotes tumor cell progression through the Hippo pathway. Furthermore, downregulation of TPM2 activates ELANE of neutrophils to facilitate ERK1/2 activation, thus enhancing IL1β and IL8 secretion for chemoattraction of more neutrophils to tumor microenvironment. The new studies identify an accomplice role for the interaction between TPM2 and ELANE in promoting LUAD progression and provide potential strategies in the prevention and/or treatment of LUAD and other cancers.

## Introduction

Lung adenocarcinoma (LUAD) remains one of the most serious human health threats globally, being responsible for more than 700,000 deaths annually. Most patients have still been diagnosed at an advanced stage, although early detection of LUAD keeps increasing [1]. Therefore, elucidating the underlying mechanisms of the malignant progression of LUAD should improve prevention and treatment for LUAD. In recent years, neutrophils are also being recognized as a part of the immune reaction to modulate tumor growth and metastatic progression [2, 3]. However, the exact role of neutrophils in tumor progression has been a matter of debate as neutrophils were shown to possess both pro- and anti-tumor properties [4–6]. Importantly, many studies in recent years have shown that patients with various cancer types, including LUAD, often exhibit increased neutrophil-to-lymphocyte ratio as an independent predictor of mortality [7]. Immune cell deconvolution identified tumor-infiltrating neutrophils as the immune population with the strongest correlation with poor outcomes across 25 cancer types, including LUAD [8]. Numerous preclinical studies uphold neutrophils as tumor accomplices, promote cancer progression and metastasis by regulation of tumor survival and migration, immune response, and angiogenesis [9, 10]. However, little is known about the therapeutic targets of neutrophils that promote the malignant progression of LUAD.

Neutrophil elastase (ELANE), a major protease in the primary granules of neutrophils, is involved in microbicidal activity [11]. Dysregulated ELANE activity also contributes to a variety of pathological processes, including chronic obstructive pulmonary disease (COPD) [12], pulmonary fibrosis [13], and atherosclerosis [14]. Moreover, studies have shown that ELANE is also an important mediator of tumor progression [15], ELANE expression and activity in human breast tumors is associated with disease recurrence and metastasis [16]. ELANE activity can be as high as three- to five-fold greater in lung cancer patients compared to those with COPD [17], driving inflammation and creating an ideal microenvironment for cancer progression in the lung [18], colorectal [19], gastric [20], and head and neck cancers [21]. Early studies with murine cancer models in lung [22], colon [23], and Pancreatic cancer [24] have suggested that ELANE could be critical in tumor initiation and growth. Recent reports suggested that cancer cells can also induce NETosis, the NET-forming process of neutrophils, to support tumor progression and metastasis [25, 26]. However, little is known about the mechanism by which lung cancer cells activate neutrophils in the lung cancer tumor microenvironment via ELANE. However, the precise mechanism through which lung cancer cells activate neutrophils within the microenvironment of lung cancer tumors via ELANE remains largely elusive.

The tropomyosin 2 (TPM2) gene is recognized for its involvement in various rare myopathies [27]. In normal physiological processes, tropomyosin is essential for regulating muscle contraction through its interaction with the actin and troponin complexes [28]. Simultaneously, TPM2 plays vital roles in regulating cell proliferation, migration, apoptosis, vesicle transport, and cytokinesis [29]. In malignant tumors, TPM2 may act as a tumor suppressor gene or a tumor promoter gene. The abnormal expression of TPM2 may result in tumorigenesis and tumor development. As a tumor suppressor gene, TPM1 was downregulated in several different types of cancers and suppressed cancer progression, including lung cancer [30] and gastric cancer [31]. Inversely, TPM2 has been reported as an oncogene. It has been reported that TPM2 expression is up-regulated in LUAD tissues, which is associated with poor prognosis in LUAD patients [32]. However, little is known about the mechanism of how TPM2 activates neutrophils to promote LUAD progression. Here, we report that downregulation of TPM2 increases neutrophil infiltration into the tumor site by regulating ELANE expression on neutrophils, thus promoting the progression of LUAD.

## Results

### TANs were associated with the malignant progression of lung adenocarcinoma

**Fig. 1:**
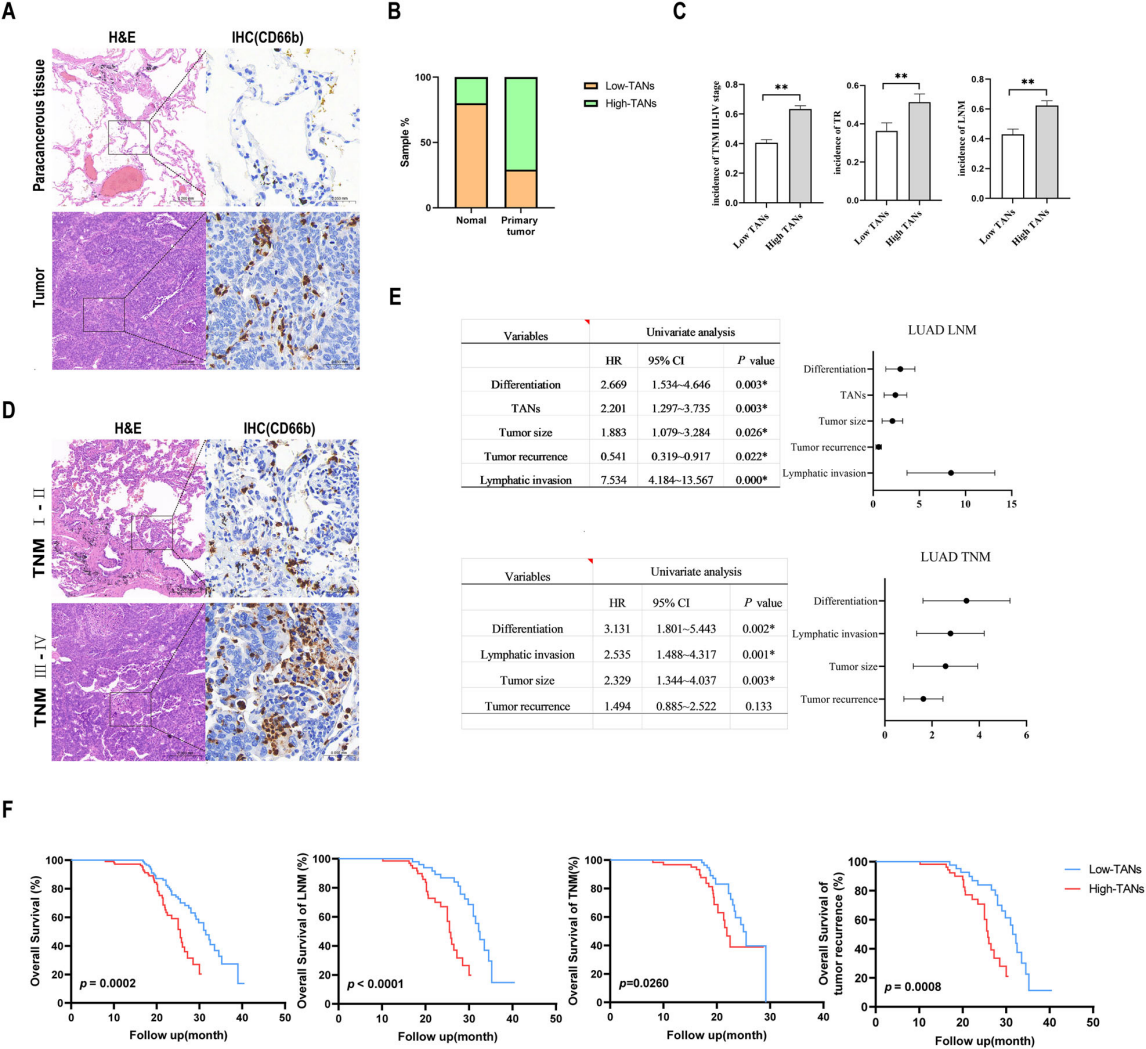
TANs were associated with the malignant progression of lung adenocarcinoma. HE and IHC were used to detect the density of TANs in LUAD and para-cancerous normal tissues (**A**), and comparisons of TANs density in all tumors and para-cancerous normal tissues (**B**). **C** The incidence of TNM III–IV stage, lymph node invasion, and tumor recurrence were compared between the high- and low-TANs groups. **D** HE and IHC were used to detect the expression of TANs in cancer tissues from LUAD patients at TNM I–II and III–IV stages. **E** Multivariate analysis was used to analyze the independent risk factors for Lymph node metastasis and TNM III–IV stage in LUAD patients. **F** Overall survival of patients with LUAD by TANs infiltration density and overall survival of LUAD patients with lymph node metastasis, TNM III–IV stage and recurrence by TANs infiltration density analyzed using Kaplan–Meier plot. *p* values were obtained by repeated measures two-tailed unpaired *t*-test; **p < 0.01; *p < 0.05; ns not significant.

To investigate the functional role of tumor-associated neutrophils (TANs) in the progression of LUAD, the clinicopathologic data of the enrolled 230 patients with LUAD were reviewed retrospectively. As shown in Fig. 1A, neutrophils could be detected in almost all LUAD cancer tissues. Based on the median TAN number of 76 per non-overlapping high-power field in primary tumor tissues, these patients were divided into high- and low-TANs groups [33], and 47.4% of cases constituted the high-TANs group (Table 1). The proportion of high-TANs groups in the primary tumor was higher than in the para-cancer tissue (Fig. 1B). High TAN levels were associated with lymph node metastasis (LNM) (*p* = 0.003), TNM III–IV stage (*p* = 0.001), tumor size (*p* = 0.026) and tumor recurrence (TR) (*p* = 0.022) (Table 1). Importantly, the incidence of TNM III–IV stage (*p* < 0.001), TR (*p* < 0.001), and LNM (*p* < 0.001) in the high-TANs group was much higher than that in the low-TANs group respectively (Fig. 1C). As shown in Fig. 1D, more TANs were infiltrated in TNM III–IV stage cancer tissues than in TNM I–II stage cancer tissues. The multivariate analysis indicated that poorly differentiated, high TANs, large tumor size, lymphatic invasion, and TR are the independent risk factors for LNM. Furthermore, poorly differentiated, lymphatic invasion and large tumor size are the independent risk factors for TNM III–IV stage (Fig. 1E).

**Table 1:** The relationship between the expression of TANs density and clinicopathological parameters in LUAD.

Clinicopathologic features	TANs		*χ*^2^	*P*
	Low (*n* = 121) (%)	High (*n* = 109) (%)		
Sex				
Male	85	78	0.048	0.827
Female	36	31		
Age				
≤60	67	49	2.49	0.115
>60	54	60		
Lymphatic invasion				
Yes	62	48	1.192	0.275
No	59	61		
Differentiation				
G1,G2	58	50	0.098	0.754
G3	63	59		
Lymph node metastasis				
Yes	52	68	8.658	0.003*
No	69	41		
TNM stage				
I, II	49	69	11.939	0.001*
III, IV	72	40		
Tumor size (d/cm)		70		
<4 cm	89	65	5.023	0.026*
≥4 cm	32	44		
Tumor recurrence				
Yes	44	56	5.259	0.022*
No	77	53		

*P* value was estimated by the Chi-square test.

*P < 0.05 was considered to be statistically significant.

To further validate the effect of TANs infiltration on patient survival, patients were followed up. Kaplan–Meier survival analysis revealed that TANs infiltration density in tumor tissues was negatively correlated with the overall survival of LUAD patients (*p* = 0.0002). In LNM (*p* < 0.0001), TNM stage III and IV (*p* = 0.026), and TR (*p* = 0.0008) with LUAD patients, the overall cancer-related survival in the high-TANs density group was significantly reduced.

### LUAD patient neutrophils interact with tumor cells to promote tumor progression

To investigate the molecular mechanism underlying TANs promoting the progression of LUAD, the effects of neutrophils on the proliferation and invasion of tumor cells were assayed. First, we utilized flow cytometry (FCM) to isolate and purify peripheral blood neutrophils from healthy donors (H-neu) or patients with advanced LUAD (A-neu) (Supplementary Fig. 1A, B). To explore the difference in the distribution of H-neu and A-neu subsets in peripheral blood, we examined the specific surface markers of neutrophils. Our results showed that LUAD patients with distant metastasis had decreased CD10 and CD62L expression, and increased CD11b expression with neutrophils compared with healthy donors (Supplementary Fig. 1C, D). Other studies have shown that compared with healthy donor peripheral blood, CD10- neutrophils are significantly increased in patients with non-Hodgkin’s lymphoma, and CD10- neutrophils may be associated with disease progression and poor prognosis [34]. CD62L low neutrophils accumulate in the lung premetastatic niche of breast cancer and capture tumor cells by producing important NETs [35], CD11b is involved in neutrophil adhesion and migration, and the phenotype of TAN is CD11b upregulation and CD62L reduction [36].

**Fig. 2:**
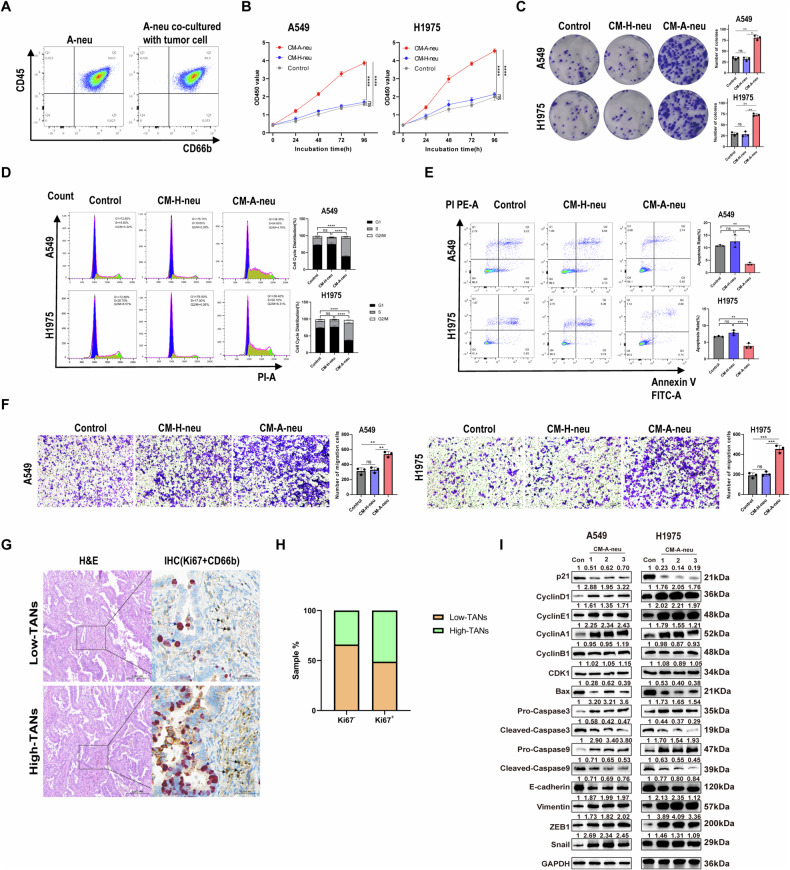
LUAD patients' neutrophils interact with tumor cells to promote tumor progression. **A** The purity of neutrophils co-cultured with or without A549 cells was detected by flow cytometry (FCM). After A-Neu or H-Neu co-culture with A549 or H1975 cells. Subsequently, these neutrophils were harvested, and their conditioned mediums (CMs) were applied to treat fresh A549 or H1975 cells to detect differences in proliferation (**B**), growth (**C**), cell cycle (**D**), apoptosis (**E**), and invasion (**F**) (100×). **G** HE and Double IHC staining were used to detect the density of TANs in LUAD tissues with high and low ki67 expression. DAB-stained CD66b was brown, GBI-stained Ki67 was red. **H** Comparisons of TANs density in LUAD tissues with high and low ki67 expression. **I** CM-A-Neu or CM-H-Neu were applied to treat fresh A549 or H1975 cells to detect cell cycle, apoptosis, and EMT-maker protein levels. *p* values were obtained by repeated measures two-tailed unpaired *t*-test; ****p < 0.0001; ***p < 0.001; **p < 0.01; *p < 0.05; ns not significant.

Neutrophils isolated from LUAD patients were then co-cultured with or without A549 cells for 12 h. FCM analyses showed that this co-culture exerted no remarkable influence on purity neutrophils (Fig. 2A). Next, the tumor-treated neutrophils from healthy donors (H-neu) or neutrophils from patients with advanced LUAD (A-neu) were cultured for an additional 6-h, the conditioned medium of A-neu (CM-A-neu) or H-neu (CM-H-neu) was collected. Notably, the CMs from tumor-treated A-neu (CM-A-neu) significantly increased the proliferation of A549 or H1975 cells by CCK-8 assay and Colony formation assay (Fig. 2B, C). Cell cycle assay showed that CM-A-neu promoted cell cycle progression in A549 and H1975 cells by regulating G1/ S phase transition (Fig. 2D). In the apoptosis assay, CM-A-neu significantly decreased the number of A549 or H1975 cells in apoptosis (Fig. 2E). Furthermore, CM-A-Neu significantly promoted invasion of A549 or H1975 cells (Fig. 2F). However, the A549 and H1975 cell lines were not affected by the CMs from tumor-treated H-neu (CM-H-neu) regulation. Next, the Ki67 status of the LUAD specimen and its correlation with TANs abundance were evaluated. Ki67 expression in tumor tissues was increased in high-TANs group compared with that in the low-TNAs group (Fig. 2G). TANs were infiltration in low levels in more than half of the tumor tissues with low expression of ki67, but most of the tumor tissues with high expression of ki67 showed high levels of TANs infiltration (Fig. 2H). Furthermore, as shown in Table 2, Spearman’s rho coefficient revealed that ki67 expression was positively correlated with TANs infiltration in OSCC (*p* = 0.03).

**Table 2:** The relationship between ki67 expression and TANs density in LUAD tissues.

ki67 expression	TANs density		Spearman’s rho coefficient test	
	Low	High	*r*	*P* value
Low	35	18	0.217	0.03*
High	22	25		

*P* value was estimated by the Spearman’s test.

*P < 0.05 was considered to be statistically significant.

To further explore the mechanism of the effect of CM-A-neu on the cell proliferation and Invasion, we detected the expression of the cell cycle, Apoptosis, and EMT-related proteins in A549 or H1975 cells treated with CM-A-neu for 24 h, respectively. Interestingly, the data revealed that P21 decreased and Cyclin A1, Cyclin D1, and Cyclin E1 proteins significantly increased after treatment, whereas the expression of Cyclin B1 and CDK1 proteins appeared to be unaffected (Fig. 2I). Studies have shown that Cyclin A1, Cyclin D1 and Cyclin E1 are the key factors regulating the transition from G0/G1 to S phase of the cell cycle, while the activation of CyclinB-CDK1 induces the late G2/M transition promoting complex/cell cycle body (APC/C), which is responsible for the final cell division. It plays a key regulatory role in the M phase. Therefore, our results indicated that CM-A-neu promoted cell cycle progression in A549 and H1975 by regulating G1/S phase transition. In addition, Bax, Cleaved-caspase 3, and Cleaved-caspase 9 protein were decreased. E-cadherin expression was inhibited whereas Vimentin, ZEB1, and Snail were increased in these tumor cells after treatment with CM-A-neu (Fig. 2I). These results suggest that crosstalk of CM-A-neu with tumor cells promotes tumor cell proliferation, anti-apoptosis and EMT, which in turn promotes tumor progression and invasion.

### ELANE from TANs is associated with poor prognosis in LUAD patients

**Fig. 3:**
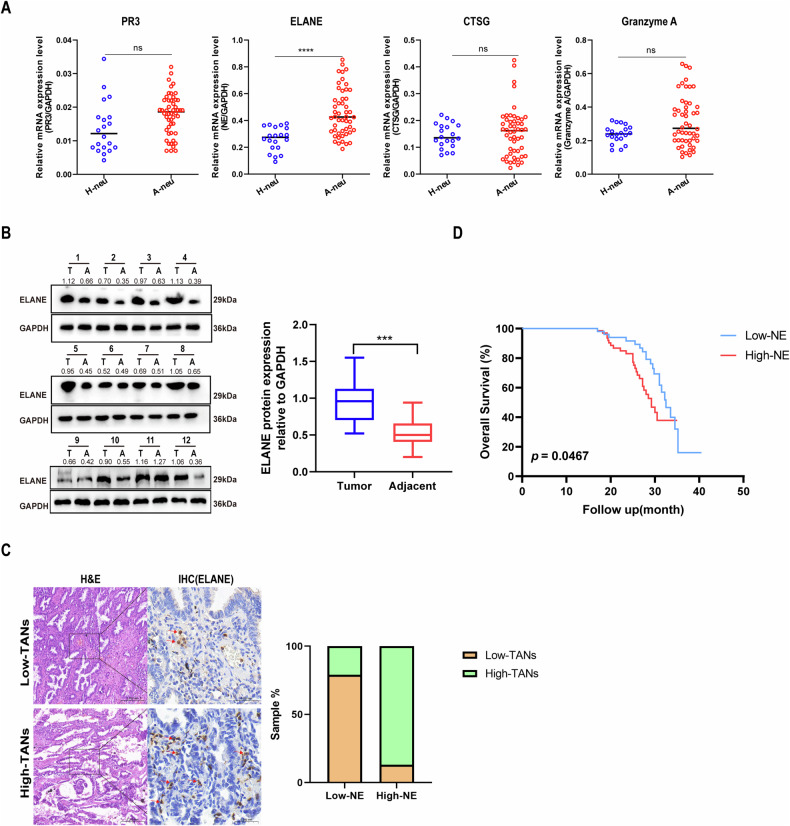
ELANE from TANs is associated with poor prognosis in LUAD patients. **A** The mRNA levels of PR3, ELANE, CTSG, and Granzyme B in peripheral blood neutrophils from 50 LUAD patients and 20 healthy donors were analyzed by QRT-PCR. **B** Western blot was used to analyze the expression of ELANE protein in 12 pairs of LUAD tissues and adjacent tissues. **C** The expression level of ELANE in high- and low-TANs group was detected by IHC, comparisons of TANs density in LUAD tissues with high and low ELANE expression. **D** Overall survival of patients with LUAD by ELANE expression level analyzed using Kaplan–Meier plot. *p* values were obtained by repeated measures two-tailed unpaired *t*-test; ****p < 0.0001; ns not significant.

Serine proteases cathepsin G (CTSG) [37], proteinase 3 (PR3) [38], neutrophil elastase [39] (ELANE), and Granzyme A [40] are proteolytic enzymes released outside the cell during neutrophil degranulation to form an extracellular snare NET. To identify which factor is responsible for tumor progression induced by CM-A-neu, we analyzed the mRNA levels of several neutrophil-derived CTSG, PR3, ELANE, and Granzyme A on peripheral blood neutrophils from 50 LUAD patients and 20 healthy donors. These proteins are usually proteolytically processed after synthesis within the cells and stored in the azurophil granules in active forms until secretion by granule exocytosis when neutrophils are stimulated. We found that the mean ELANE mRNA level was 1.75-fold higher in the peripheral blood of LUAD patients compared with healthy donors, but there was no difference in CTSG, PR3, or Granzyme A (Fig. 3A). Subsequently, we detected the CTSG, PR3, ELANE, and Granzyme A protein levels in twelve paired LUAD tissues and adjacent tissues. The results showed that ELANE protein levels were significantly higher in LUAD tissues compared with those in adjacent tissues (Fig. 3B), suggesting that ELANE was overexpressed in the LUAD tissues. However, the protein expression of CTSG, PR3, and granzyme A did not differ between cancer and adjacent tissue (Supplementary Fig. 2).

To further analyze the relationship between the expression of ELANE and the prognosis of LUAD patients, we analyzed the expression of ELANE in the cancer tissues and corresponding adjacent tissues of 230 patients by immunohistochemistry (IHC). The results showed that the higher the density of neutrophil infiltration in LUAD cancer tissues, the higher the expression level of ELANE, and ELANE was highly expressed in more than half of the high-TANs groups, but ELANE expression was low in most of the low-tans groups (Fig. 3C). In addition, we also found that high ELANE expression on neutrophils linked to shortened overall survival of the LUAD patients (Fig. 3D).

### TANs-derived ELANE promotes tumor progression by activating the Hippo pathway in tumor cells

**Fig. 4:**
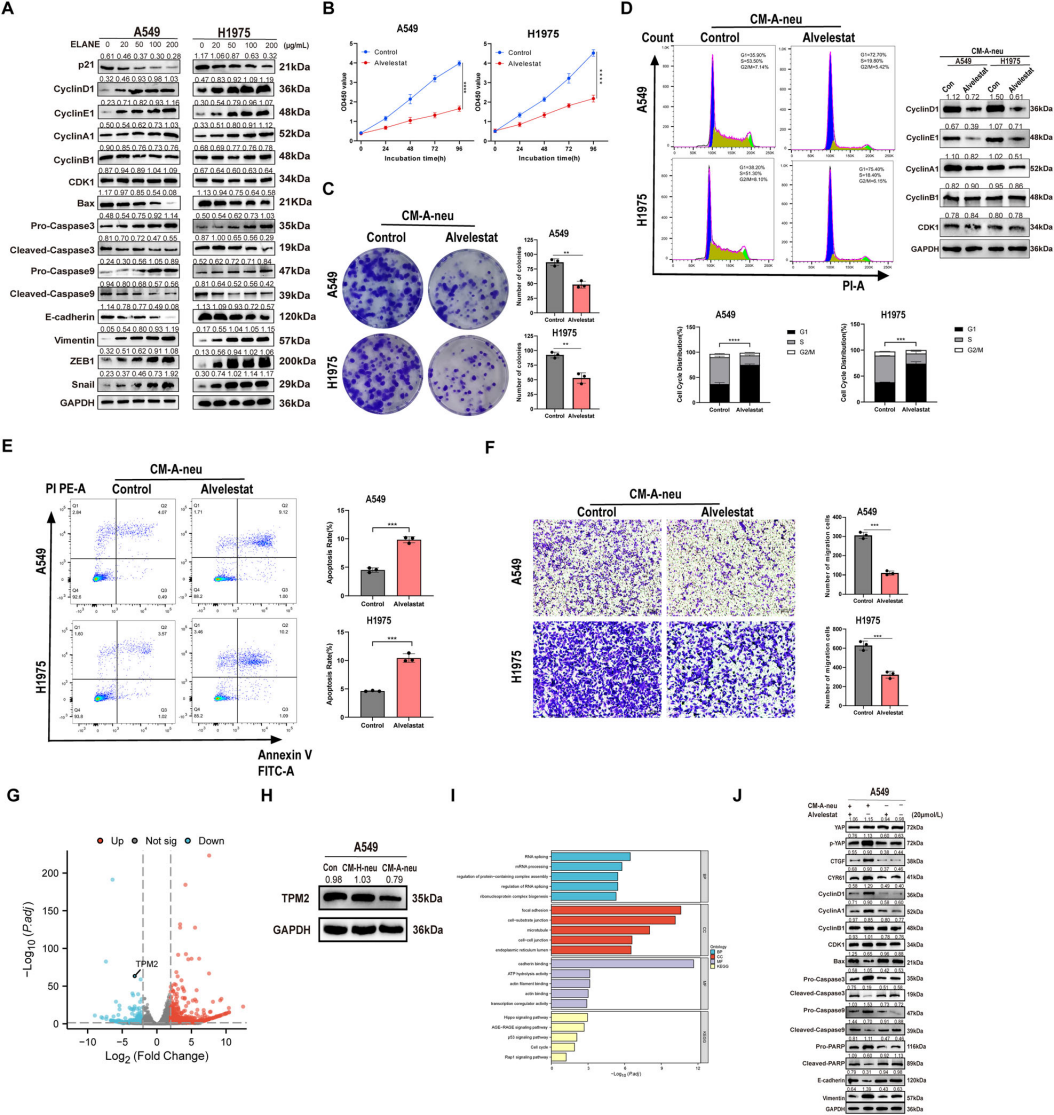
ELANE enhances tumor progression by acting on the tumor cell to promote the activation of the Hippo pathway in tumor cells. **A** A549 and H1975 cells were treated with different concentrations of exogenous ELANE recombinant protein to detect the cell cycle, apoptosis, and EMT-producing protein levels. **B**–**F** CM-A-Neu-mediated tumor cells A549 and H1975 were treated with or without ELANE inhibitor HY-15651. The differences of proliferation (**B**), growth (**C**), cell cycle (**D**), apoptosis (**E**), and invasion (**F**) (100×) were detected. **G** Volcano map representing the differentially expressed proteins in A549 cells treated with CM-A-Neu vs A549 cells treated with CM-H-Neu. **H** The TPM2 protein level of A549 cells treated with CM-A-Neu and CM-H-Neu was detected. **I** GO and KEGG enrichment analysis based on the 362 downregulated proteins in A549 cells treated with CM-A-Neu. **J** CM-A-Neu-mediated tumor cells A549 were treated with or without ELANE inhibitor HY-15651 to detect the Hippo pathway, Cycle, apoptosis, and EMT-maker protein levels. *p* values were obtained by repeated measures two-tailed unpaired *t*-test; ****p < 0.0001; ***p < 0.001; **p < 0.01; *p < 0.05.

To elucidate the specific molecular mechanism of tumor progression induced by CM-A-neu. We found that ELANE increased the expression of cyclin protein, while decreased the expression of apoptotic proteins Bax, Cleaved-caspase 3, and Cleaved-caspase 9 in A549 or H1975 cells in a dose-dependent manner. Moreover, ELANE increased the expression of EMT-related proteins in LUAD cells in a dose-dependent manner (Fig. 4A). Furthermore, blockade of ELANE with neutralizing antibody, Alvelestat, could reverse the enhancement of proliferation, anti-apoptosis, and invasion of tumor cells induced by CM-A-neu (Fig. 4B–F).

RNA sequencing (RNA-seq) was performed to analyze the gene expression profile of A549 cells after 24 h co-culture with peripheral blood neutrophils from advanced LUAD patients (CM-A-Neu) or peripheral blood neutrophils from healthy donors (CM-H-Neu). Multiple differentially expressed genes (DEGs) were identified, including 784 up-regulated genes(Supplementary Table 1) and 637 downregulated genes (Supplementary Table 2), respectively. Volcano map showed TPM2 was significantly downregulated in CM-A-Neu-treated A549 cells compared with CM-H-Neu-treated A549 cells (Fig. 4G, Supplementary Table 3). Similarly, western blot assay results found that the protein level of TPM2 was significantly downregulated in A549 cells treated with CM-A-Neu (Fig. 4H). Studies have reported that TPM2 is low expressed in lung cancer tissues and is associated with poor prognosis [32]. Furthermore, we selected 362 downregulated proteins to perform Kyoto Encyclopedia of Genes and Genomes (KEGG) enrichment analysis of cellular components and found that several pathways were enriched, such as the Hippo pathway, AGE-RAGE signaling pathway, p53 pathway, and Rap1 pathways, among them, Hippo pathway has the most significant difference (Fig. 4I). GO analysis showed that these downregulated proteins in A549 cells treated with CM-A-Neu were mainly involved in the biological process of mitosis, the role of cell junction cytoskeleton and the activation of some transcription factors (Fig. 4I).

The Hippo pathway is a crucial regulator of organ size, tissue homeostasis, and tumorigenesis [41, 42]. Downstream of Hippo signaling, YAP/TAZ mainly regulates the expression of cell proliferation, differentiation, and survival-related target genes CYR61 and CTGF, and then affects the growth and tumorigenesis of organs [43]. To further determine whether the Hippo pathway is activated under ELANE stimulation, blockage of ELANE with Alvelestat-treated A549 cells was analyzed. It was found that the p-YAP, CYR61, and CTGF were significantly decreased upon Alvelestat treatment. Furthermore, Alvelestat could reverse tumor cell proliferation, anti-apoptosis, and the expression of EMT-related proteins mediated by CM-A-neu (Fig. 4J). These findings proved that TANs-derived ELANE induced the activation of tumor cells and promoted the LUAD progression through the Hippo pathway.

### Downregulation of TPM2 enhances tumor progression by acting on p38-MAPK pathway to promote ELANE expression in neutrophils

**Fig. 5:**
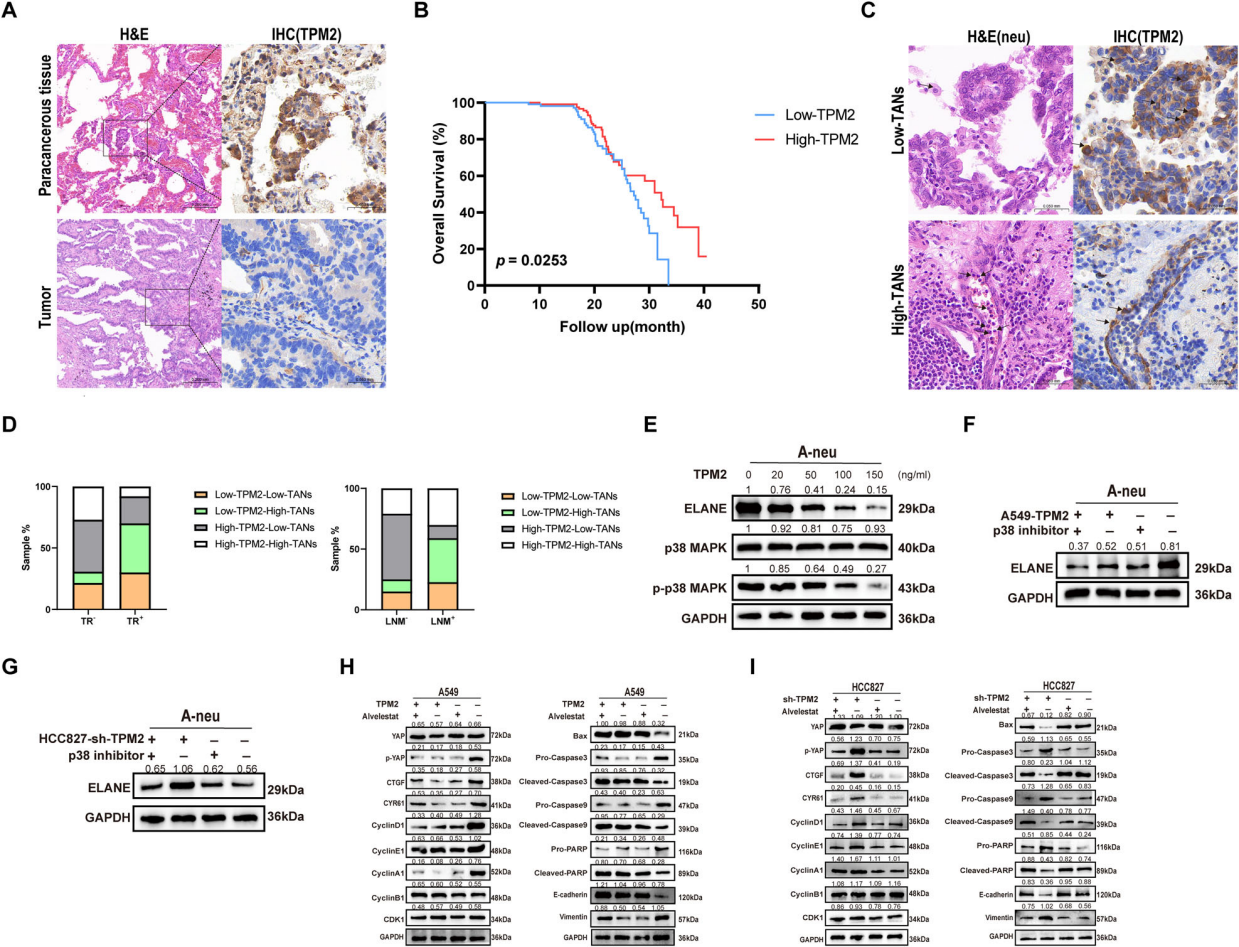
Downregulation of TPM2 enhances tumor progression by acting on p38-MAPK pathway to promote ELANE expression in neutrophils. **A** The expression of TPM2 in LUAD and para-cancerous normal tissues was detected. **B** Overall survival of patients with LUAD by TPM2 expression analyzed using Kaplan–Meier plot. **C** HE was used to detect TANs infiltration density in LUAD tissues. IHC was used to detect the expression level of TPM2 and TANs infiltration density in LUAD tissues. **D** The expression of TPM2 and the infiltration density of TANs in LUAD patients with or without lymph node metastasis and tumor recurrence were compared. **E** A-Neu were treated with different concentrations of exogenous TPM2 recombinant protein to detect ELANE, p38/MAPK, and p-p38/ MAPK protein levels. **F**, **G** TPM2-overexpressed A549 and TPM2-knockdown HCC827 and/or p38/ MAPK inhibitor SB202190 were co-cultured with A-Neu to detect ELANE expression levels. **H**, **I** TPM2-overexpressed A549 and TPM2-knockdown HCC827 were treated with or without ELANE inhibitors to detect Hippo pathway, Cycle, apoptosis, and EMT-maker protein expression levels.

We next investigated the potential molecular signaling responsible for tumor cell-mediated upregulation of ELANE in neutrophils during their crosstalk. TPM2 has been reported to be involved in middle to advanced tumor progression in a variety of cancer types, including LUAD [32]. Notably, TPM2 expression was lower in LUAD tissue. Therefore, we proposed that tumor-derived transforming downregulation of TPM2 may be involved in ELANE upregulation in neutrophils. IHC assay results showed that TPM2 was less expressed in human LUAD than in para-cancer tissue (Fig. 5A). Kaplan–Meier survival analysis revealed that downregulation of TPM2 expression was associated with the shorter overall survival in LUAG patients (Fig. 5B). Moreover, tumor tissue from patients with downregulation of TPM2 expression had a higher TANs infiltration density (Fig. 5C). Spearman’s rho coefficient revealed that TPM2 expression was negatively correlated with TANs infiltration in OSCC (*p* = 0.019) (Table 3). Moreover, we found that downregulation of TPM2 expression and high-TANs infiltration density group was detected in more than 36.3% of LUAD cancer tissues with LNM, but only 10% in those without LNM. Similarly, downregulation of TPM2 expression and high-TANs infiltration density group were detected in more than 40.0% of LUAD cancer tissues with TR, but only 9.2% in those without TR (Fig. 5D).

**Table 3:** The relationship between TPM2 expression and TANs density in LUAD tissues.

TPM2 expression	TANs density		Spearman’s rho coefficient test	
	Low	High	*r*	*P* value
Low	21	26	−0.234	0.019*
High	36	17		

*P* value was estimated by the Spearman’s test.

*P < 0.05 was considered to be statistically significant.

We conducted a deeper investigation into how TPM2 regulates ELANE expression. We particularly focused on p38/MAPK, since previously p38/MAPK were shown to be involved in leukocyte production of ELANE in neutrophils [44, 45]. We found that Exogenous TPM2 could down-regulate the expression of ELANE, phosphorylated p38 MAPK (p-p38 MAPK) in neutrophils in a dose-dependent manner (Fig. 5E). Based on endogenous expression of TPM2 in LUAD cell lines (Supplementary Fig 3A), we constructed A549 cells stably overexpressing TPM2 (Supplementary Fig. 3B), which have a low basal expression of TPM2, we knocked down TPM2 by shRNAs in HCC827 cells (Supplementary Fig. 3C), which have high basal expression of TPM2. After treatment with SB202190, a p38 mitogen-activated protein kinase (MAPK) pathway inhibitor, the expression of ELANE mediated by downregulation of TPM2 expression was reversed, suggesting that TPM2 regulates ELANE expression through p38/MAPK signaling pathway (Fig. 5F, G). Furthermore, treatment with A549 cells with TPM2 overexpression or ELANE receptor inhibitor Alvelestat showed that Alvelestat reversed activation of the low-expressed TPM2-mediated hippo pathway, thereby reversing tumor cell cycle, apoptosis, and expression of EMT-associated proteins (Fig. 5H). Conversely, the knockdown of TPM2 in HCC827 cells activated the hippo pathway through the upregulation of ELANE (Fig. 5I). Collectively, these results strongly suggest that tumor cells increase the level of ELANE by down-regulating the interaction between TPM2 and neutrophils and through the p38/MAPK pathway, ELANE mediates tumor cell activation and promotes tumor progression.

### Downregulation of TPM2 promotes neutrophil recruitment to tumor sites by activating the ELANE-ERK1/2-IL1β/IL8 axis of neutrophils

**Fig. 6:**
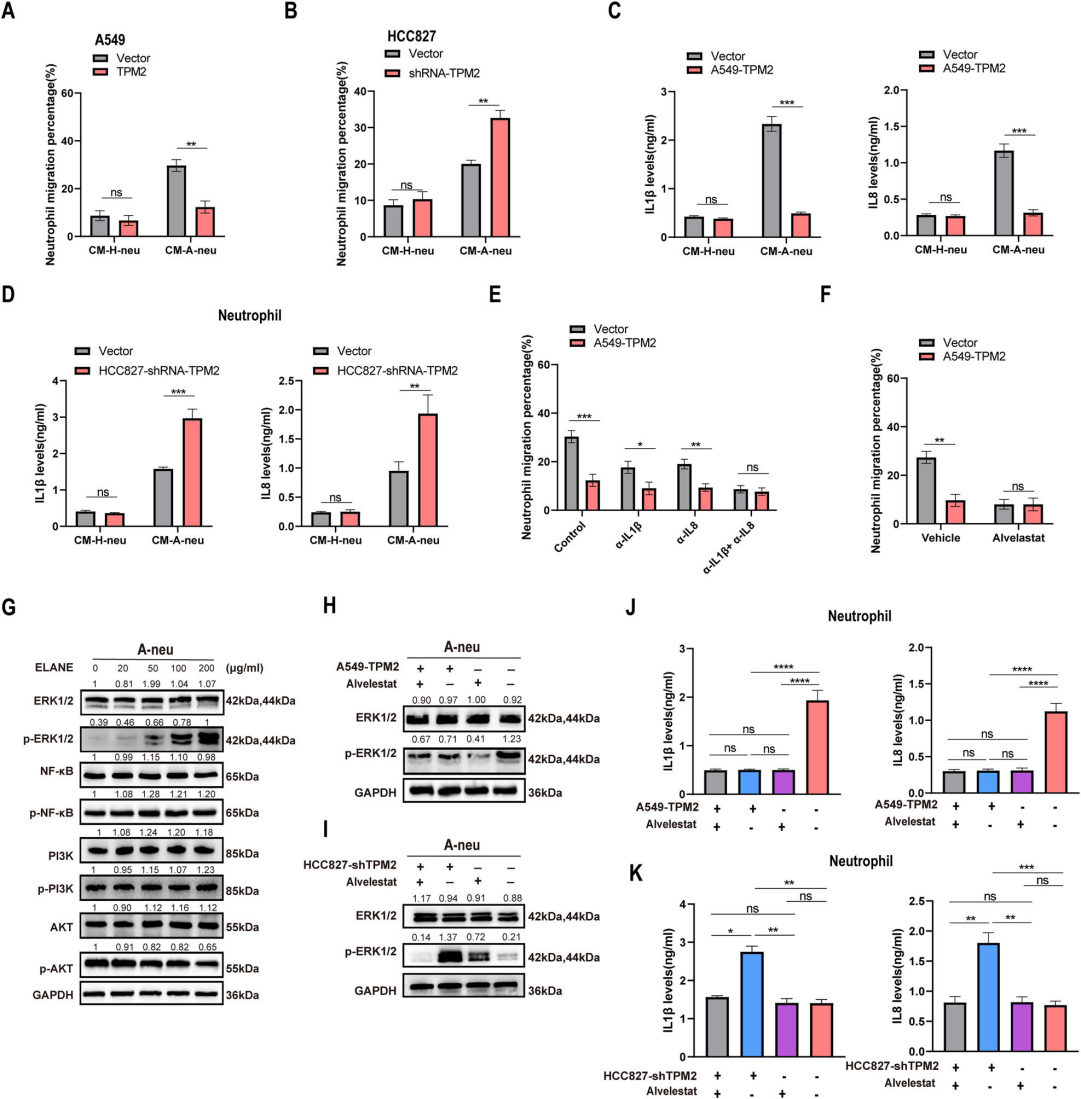
Downregulation of TPM2 promotes neutrophil recruitment to tumor sites by activating the ELANE-ERK1/2-IL1β/IL8 axis of neutrophils. **A**, **B** Migration of Peripheral blood-derived neutrophils recruited by conditioned medium of neutrophils obtained from A-neu and H-neu pretreated with TPM2-overexpressed A549 cells or TPM2-konckdown HCC827 cells. **C**, **D** A-neu and H-neu IL1β and IL8 secretion levels after pretreated with TPM2-overexpressed A549 cells or TPM2-konckdown HCC827 cells. **E** Migration of Peripheral blood-derived neutrophils recruited by CM-A-neu+ TPM2 with and/or IL1β and IL8-neutralizing antibodies. **F** Migration of Peripheral blood-derived neutrophils recruited by CM-A-neu+ TPM2 with ELANE inhibitor Alvelestat. **G** The expression of ERK, JNK1, NF-kB, PI3K, and Akt pathway proteins were detected after neutrophils were pretreated with different concentrations of ELANE. **H**, **I** After pretreatment of neutrophils with TPM2-overexpressed A549 cells/TPM2-konckdown HCC827 cells and/or 20 μmol/l ELANE inhibitor (Alvelestat) to detect p-ERK1/2 and ERK1/2 expression. **J**, **K** Protein levels of IL1β and IL8 in A-neu after pretreated with TPM2-overexpressed A549 cells or TPM2-konckdown HCC827 cells combined with 20 μmol/l ELANE inhibitor (Alvelestat). *p* values were obtained by repeated measures two-tailed unpaired *t*-test; ****p < 0.0001; ***p < 0.001; **p < 0.01; *p < 0.05; ns not significant.

Next, we interrogated how TPM2 affects neutrophil recruitment to the tumor microenvironment. Intriguingly, TPM2 overexpression in A549 changed the capability of the CM-A-neu mentioned to directly attract neutrophils, whereas it had no effect on CM-H-neu (Fig. 6A). In contrast, TPM2 knockdown in HCC827 promotes chemotaxis of neutrophils by CM-A-neu (Fig. 6B). This suggests that TPM2 may act on tumor-derived neutrophils to regulate some chemokines. Previous studies have shown that IL1β [46], IL5 [47], IL6 [48], IL8 [49], GM-CSF [47], and TNF-ɑ [50] are common neutrophil chemokines involved in neutrophil aggregation in the tumor microenvironment. Quantitative PCR and ELISA validated that TPM2 overexpression downregulated IL1β and IL8 expression among these cytokines in CM-A-neu but not in CM-H-neu (Fig. 6C, Supplementary Fig. 4A–C). Conversely, TPM2 knockdown upregulates the expression of IL1β and IL8 in cytokines in CM-A-neu (Fig. 6D, Supplementary Fig. 4D). Further, our data showed that recombinant IL1β and IL8 proteins were able to drive the chemotaxis of neutrophils in a dose-dependent manner (Supplementary Fig. 4E). In addition, administration of IL1 (a-IL1β) or IL8 (a-IL8) blocking antibody in the CM-A-neu can partially inhibited neutrophil recruitment mediated by low TPM2 expression, while simultaneous blockade of both factors completely abrogated the effect of TPM2 (Fig. 6E). These data suggested that downregulation of tumor-derived TPM2 acts on neutrophils that are already present in the vicinity of cancer cells to regulate the secretion of IL1β and IL8 and recruit more neutrophils to infiltrate the tumor microenvironment.

The aforementioned experimental results clearly showed that TAN clusters in the tumor microenvironment promote tumor progression through TPM2 interacting with ELANE. However, the molecular mechanisms underlying cluster infiltration remain unclear. We found that treatment with the ELANE inhibitor, Alvelestat, could effectively abrogate neutrophil recruitment induced by low TPM2 expression (Fig. 6F), suggesting that TPM2 regulates neutrophils through ELANE. Then we asked how ELANE regulates neutrophil chemotaxis. Previous studies demonstrated that chemokines IL1β and IL8 were shown to be downstream targets of ERK1/2 [51], NF-kB [52], and PI3K/Akt [53]. To identify signaling pathways for neutrophil chemotaxis induced by TPM2 and ELANE, we found that ELANE increased ERK1/2 expression in neutrophils in a dose-dependent manner, but had no effect on NF-kB, and PI3K/Akt pathways (Fig. 6G). Indeed, A549 cells with low expression of TPM2 increased ERK1/2 phosphorylation in human neutrophils. Such an effect of TPM2 could be abrogated by Alvelestat, supporting TPM2 regulates activation of the ERK1/2 pathway in neutrophils via ELANE (Fig. 6H). The opposite result was obtained by knocking down TPM2 in HCC827 cells (Fig. 6I). We observed that A549 cells with low TPM2 expression increased the expression of the ERK1/2 target genes il-1β and il-8 in neutrophils. Similarly, this effect of low expression of TPM2 can be eliminated by Alvelestat (Fig. 6J, Supplementary Fig. 4F). In contrast, knockdown of TPM2 promoted the expression and secretion of IL1β and IL8, which could be blocked by Alvelestat(Fig. 6K, Supplementary Fig. 4G). Collectively, these data suggested that downregulation of TPM2 enhances neutrophil chemoattraction by activating neutrophil ELANE and promoting ERK1/2-IL1β/IL8 signaling.

### Low TPM2 expression enhances tumor growth by activating neutrophils ELANE to promote neutrophil recruitment to tumor microenvironment in vivo

**Fig. 7:**
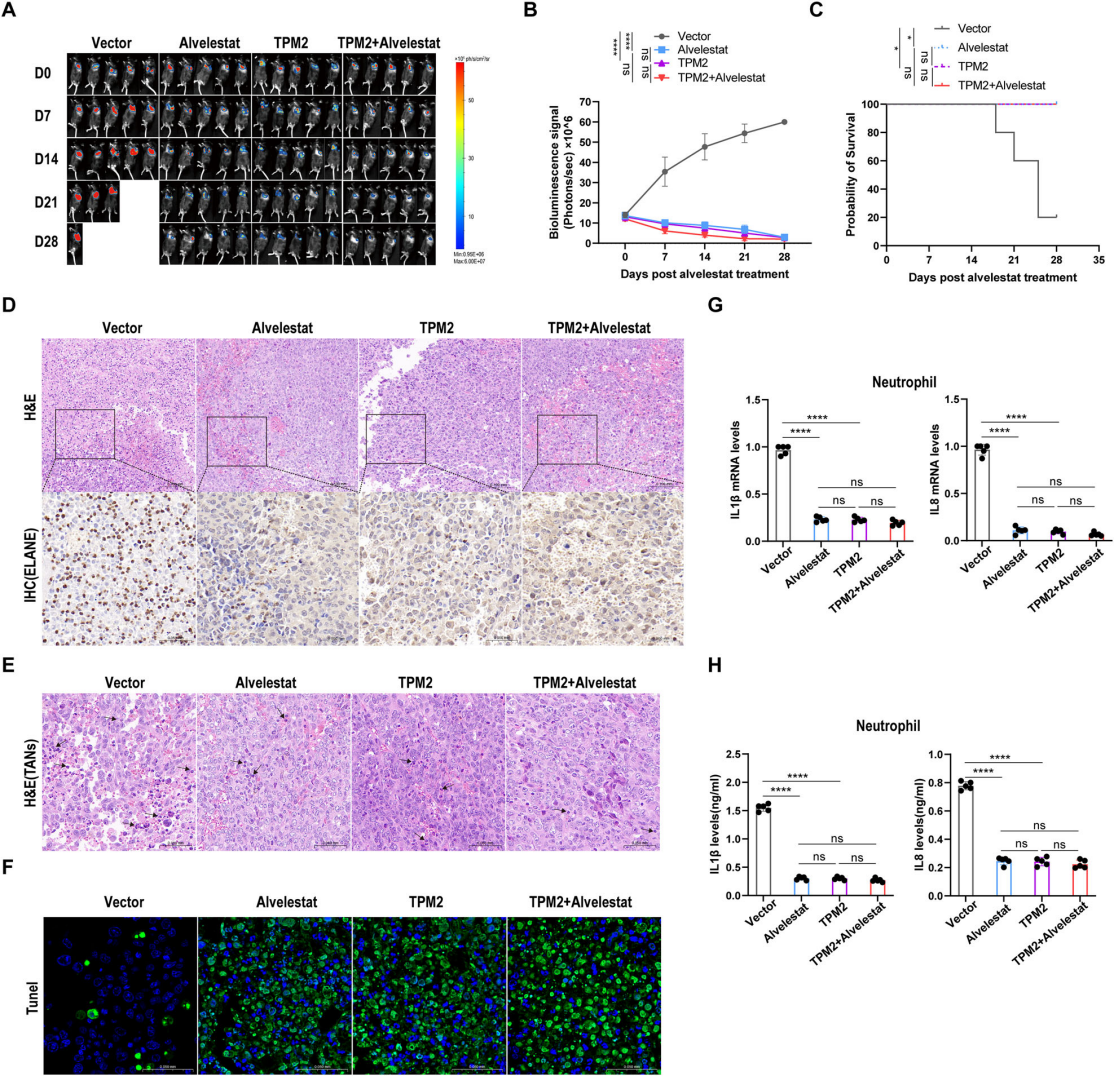
Lower TPM2 expression enhances tumor growth by activating neutrophils ELANE to promote neutrophil recruitment to tumor microenvironment in vivo. **A** The bioluminescence imaging (BLI) in PLVX, Alvelestat, TPM2, and TPM2+Alvelestat treatment groups (*n* = 5). **B** Quantitation of total body bioluminescence intensity (*n* = 5). **C** Kaplan–Meier survival curves (*n* = 5). *P* values are determined by the Log-rank (Mantel–Cox) test. **D**, **E** HE and IHC were used to detect ELANE expression and neutrophil infiltration density in C57BL/6 mouse tumor tissues. **F** The apoptosis level of the C57BL/6 mouse tumor was detected. **G**, **H** IL-1β and IL-8 mRNA and protein levels in peripheral blood neutrophils of C57BL/6 mice were detected. *p* values were obtained by repeated measures two-tailed unpaired *t*-test; ****p < 0.0001; *p < 0.05; ns not significant.

The Orthotopic (intralung) tumor models of lung cancer in C57BL/6 mice using vector-luciferized and TPM2 overexpression-luciferized-LLC cells, a mouse lung cancer cell line, were established to investigate the effects of the interaction between TPM2 in tumor cells and ELANE in neutrophils on tumorigenesis. We imaged the animals weekly to assess tumor burden. In vivo imaging results showed that lung tumors in the TPM2 overexpression group were gradually reduced compared to the vector group (Fig. 7A, B). We observed longer survival in the TPM2 group and the TPM2+Alvelestat group than in the Vector group (Fig. 7C). In addition, compared with the vector group, the Alvelestat group significantly reduced the expression of ELANE in tumors and slowed tumor growth (Fig. 7A, B, D). Further analysis showed that the Alvelestat group reduced neutrophil infiltration in tumors and increased tumor cell apoptosis (Fig. 7E, F).

Next, we interrogated how TPM2 affects neutrophil recruitment via ELANE in vivo. Quantitative PCR and ELISA detection showed that compared with the Alvelestat group, the expression levels of chemokines IL1β and IL8 of neutrophils in the Vector group were higher (Fig. 7G, H). The results suggest that low expression of TPM2 promotes tumor growth by activating neutrophil ELANE and the ERK1/2-IL1β/IL8 axis of neutrophils to promote neutrophil recruitment to the tumor microenvironment.

## Discussion

Mounting evidence established that increased TAN accumulation is a poor prognostic marker in many cancers [54]. Our comprehensive clinicopathologic investigations have demonstrated that TANs accumulate in the LUAD tumor microenvironment and promote tumor progression. We further revealed that high levels of TANs were an independent risk factor for LUAD patients and that higher TANs infiltration in LUAD was linked to LNM, TNM III–IV stage, and TR and shortened overall survival of the LUAD patients. We further revealed the difference between neutrophils from cancer patients and healthy donors in promoting tumor progression and found that neutrophils from cancer patients but not healthy donors were more advantageous in promoting tumor progression. It may be that the release of immature neutrophils or the alteration of neutrophil phenotype under the influence of the tumor microenvironment promotes tumor growth [3, 46]. In our study, we found that LUAD patients with distant metastasis had decreased neutrophil CD10 expression, increased CD11b expression, and decreased CD62L expression compared with healthy donors. The intricate relationship between neutrophils and cancer progression depends on multiple factors, such as tumor characteristics [55], endogenous influences, and the overall health status of patients [56].

The ability of neutrophils to promote tumor progression has been known for decades; however, the molecular mechanisms that mediate their recognition of tumor cells remain unknown. We provide evidence that the high expression of ELANE in TANs leads to poor prognosis of LUAD patients and this is due to the interaction of ELANE with cancer cells. Consistent with our findings, Lulla AR and Houghton AM et al. found that the amount of immunoreactive ELANE in tumor tissue is an independent prognostic indicator of patients with breast cancer and lung cancer [16, 22]. Furthermore, a specific NE inhibitor completely suppressed the growth of cancer cells transplanted into severe combined immunodeficiency mice [15]. RNA-seq analysis revealed that co-culture with neutrophils from patients with advanced LUAD induced extensive signaling pathway gene expression alterations in tumor cells, including a significant upregulation of the Hippo pathway, which suggests that tumor cells are educated to transform into active states by TANs. The Hippo signaling pathway has been associated with controlling organ size and the development of cancer [41, 43]. YAP is the chief downstream effector of the Hippo pathway that regulates the function of tumor cells. Mechanistically, we found that TANs-derived ELANE promoted tumor progression by activating the p-YAP in tumor cells. Further analysis of RNA-seq showed tumor cells activated by TANs showed downregulated expression of several cancer-related genes, including TPM2. Our results showed that TPM2 was low expressed in LUAD tissues and was a major factor for poor prognosis in LUAD patients. These findings are interesting in light of the fact that TPM2 expression is frequently downregulated in cancer where it promotes tumor growth, tumor cell migration, and metastasis [30, 31]. We hypothesized that due to the downregulation of TPM2 in tumor cells, TANs might recognize this pattern and target tumor cells. The P38/ MAPK pathway has been shown to be involved in the leukocytosis of ELANE in neutrophils [44, 45]. Indeed, our data show that the use of p38/ MAPK inhibitors indicates that downregulation of TPM2 activates and upregulates ELANE expression in neutrophils through the p38/ MAPK pathway. We further demonstrated that TPM2 expression was inversely correlated with ELANE levels in LUAD tissues. Collectively, these results strongly suggest that tumor cells increase ELANE levels through the TPM2-p38/MAPK pathway in neutrophils during their interaction, and ELANE-mediated tumor progression is through Hippo signaling activation. This detrimental cycle enhances tumor cell activation and promotes LUAD progression.

Interestingly, the depletion of neutrophils leads to the alleviation of tumor metastasis in chemically induced and spontaneous cancer models, underscoring the important potential of reducing neutrophils infiltration into the tumor microenvironment to mitigate cancer progression [57, 58]. Our data elucidate that downregulation of TPM2 was capable of activating the ELANE-ERK1/2 pathway and increasing IL1β and IL8 production. Inhibition of NE with Alvelestat blocked the ERK1/2 pathway and secretion of IL1β and IL8 by tumor cells with downregulation of TPM2. Moreover, an IL1β/IL8-neutralizing antibody abolished the enhanced chemotactic effect of downregulation of TPM2 tumor cells on TANs. Last, overexpression of tumor cells TPM2 and inhibition of neutrophil ELANE expression achieved satisfactory antineoplastic effects by reduced neutrophil infiltration of tumor microenvironment in the Orthotopic (intralung) tumor models of lung cancer. Our research provides a scientific basis for the importance of TANs in the tumor microenvironment for the progression of LUAD.

**Fig. 8:**
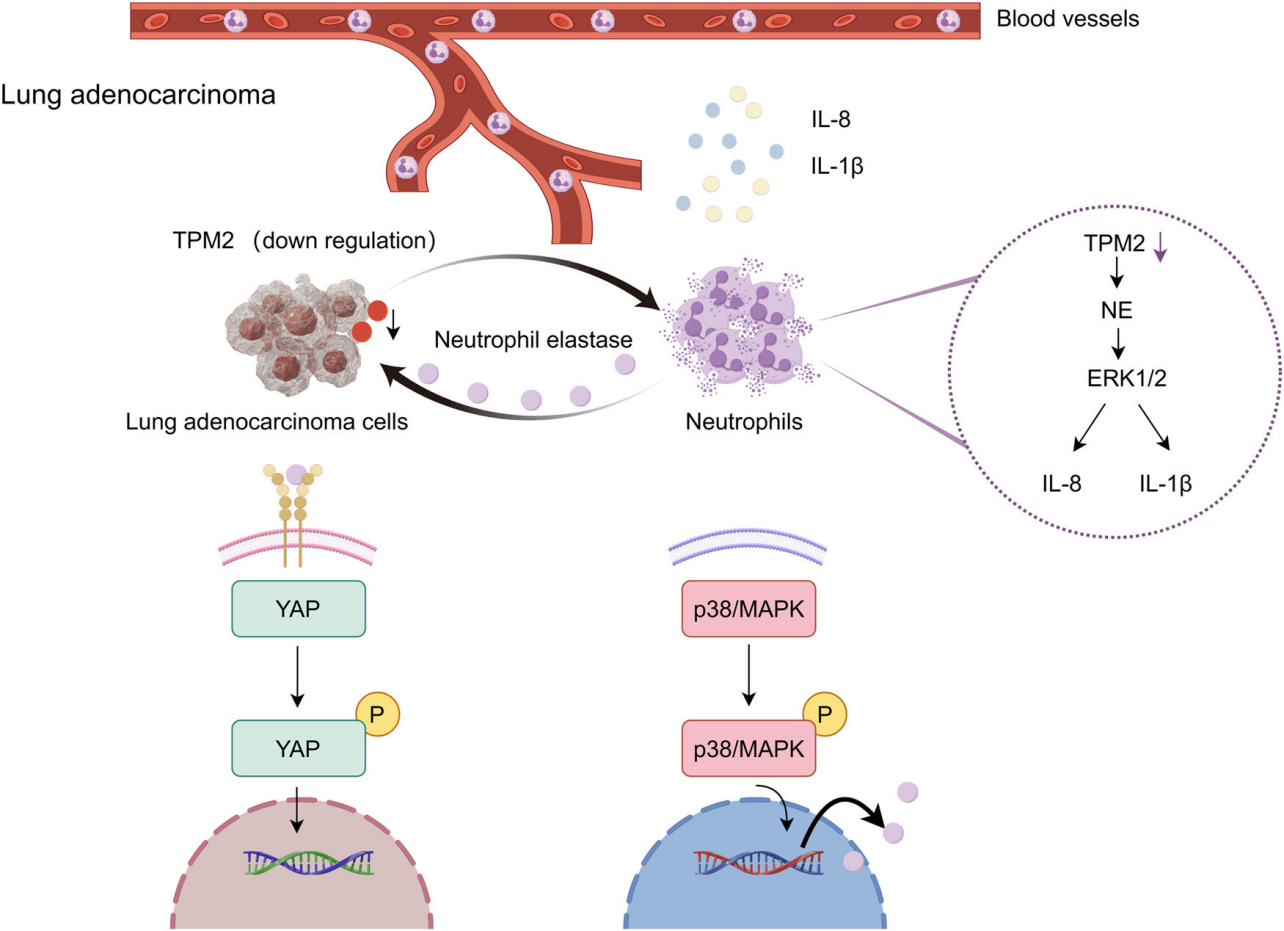
Tumor cells mediate tumor progression by down-regulating TPM2 to induce TANs to produce more ELANE.

In conclusion, our study demonstrates that tumor cells educate TANs via downregulation of TPM2 to produce more ELANE, which mediates tumor progression. This detrimental loop enhances tumor cell proliferation and invasiveness (Fig. 8), which indicates that targeting TPM2 of tumor cells and ELANE of TANs is a potential strategy to improve the prognosis of LUAD.

## Materials and methods

### Cell culture

H1975, A549 human cell lines and LLC mice cell lines were acquired from the American Type Culture Collection (ATCC, USA) and cultured in Rosewell Park Memorial Institute (RPMI)-1640 or Dulbecco’s modified Eagle’s medium (DMEM) supplemented with 10% fetal bovine serum (HyClone, Logan, UT, USA), 100 U/mL penicillin and 100 μg/mL streptomycin at 37 °C with 5% CO_2_.

### Patients

Peripheral blood samples were collected from healthy individuals (*n* = 20) and patients with LUAD (*n* = 50). A total of 230 patients with primary LUAD treated at the Shandong Provincial Hospital Affiliated with Shandong First Medical University between 2011 and 2023 participated in this study. The clinical pathological data of all the patients were complete, and no radiotherapy or chemotherapy was performed before surgery. The clinicopathological information of the patients is presented in Table 1. Cancer and para-cancer normal tissue samples were obtained during the operation, immediately immersed in 10% formalin, and subsequently made into tissue microarrays and analyzed by immunohistochemistry (IHC). Twelve patients had both tumor and adjacent tissue sufficiently large so that a portion of the tissue was immediately frozen in liquid nitrogen and stored for protein analysis. This study was approved by the Institutional Review Board of the Ethics Committee of the Affiliated Hospital of Qingdao University (SYFY WZLL20115) and performed in accordance with the guidelines of the Declaration of Helsinki.

### Immunohistochemistry (IHC)

Tissue microarrays from humans or paraffin-embedded tissue sections from mice were deparaffinized in a series of gradient ethanol baths, rehydrated, and immersed in methanol containing 0.3% hydrogen peroxide for 10 min to block endogenous peroxidase at room temperature. Subsequently, the tissue slides were heated for 30 min in a pH 6.0 antigen retrieval solution to induce antigen retrieval and then incubated overnight with a Rabbit anti-CD66b (Abcam, Cambridge, UK), Mouse anti-CD15 (Abcam, Cambridge, UK), Mouse anti-Ki67 (Abcam, Cambridge, UK), Rabbit anti-Ly6g(Abcam, Cambridge, UK), Rabbit anti-Tropomyosin 2 (Abcam, Cambridge, UK), Rabbit anti-Neutrophil Elastase (Abcam, Cambridge, UK) at 4 °C Staining was performed using a Prolink-2 Plus HRP rabbit polymer detection kit (Golden Bridge, Bothell, WA, USA) according to the manufacturer’s instructions. Meanwhile, a polymer double-staining kit (Zhongshan Golden Bridge Biotech, China) was used for staining. Staining was performed as above. The polymer horseradish peroxidase detection system (ZSGB, China) in this work used DAB and GBI for visualization and hematoxylin for nuclear counter staining. The results showed that DAB-stained CD66b was brown, GBI (Giemsa-Based Immunohistochemistry) -stained Ki67 was red, and hematoxylin-stained cell nuclei were blue. The results were evaluated based on the intensity and extent of staining by two independent pathologists (double-blinded) as described previously.

TPM2, ELANE, and ki67 staining were evaluated on the basis of a semiquantitative scoring system. The intensity score represented the average intensity of the positive tumor cells (0, none; 1, weak; 2, intermediate; and 3, strong). The proportion score represented the estimated proportion of positive tumor cells: 0: (<5%), 1: (5%-25%), 2: (26%-50%), 3: (51%-75%), and 4: (>75%). The proportion and intensity scores were then added to obtain a total score, which ranged from 0 to 7. All specimens were divided into two groups: weak expression, 0–3 points and strong expression, 4–7 points. For the CD66b+ neutrophil count, positive cells in three cylinders with a diameter of 1 mm per patient were calculated and presented as the mean value of the triplicates (cells/core). The average value of CD66b+ neutrophils in TMA was acquired, and the median of CD66b+ neutrophils of all samples was obtained as the cut-off value in subsequent analysis.

### Neutrophil isolation

Peripheral neutrophils were isolated from 20 mL whole blood samples of healthy donors or patients with LUAD using a human or mice peripheral blood neutrophil separation reagent kit (Solarbio, Beijing, China) as per the recommended protocols. The cells were washed with red blood cell lysis buffer (BD Biosciences, NJ, USA), centrifuged, and washed with PBS. The isolated neutrophils were cultured in complete RPMI1640 (GIBCO, VA, USA). To determine the purity and viability, the isolated neutrophils were washed and resuspended in stain buffer (BD Biosciences, NJ, USA) for FCM analysis (BD FACSAria II flow cytometer, BD Biosciences, Franklin Lakes, NJ, USA). FITC anti-human CD45 antibody and APC anti-human CD66b antibody were used respectively.

### Cell counting Kit-8 (CCK-8) and colony formation assays

A CCK-8 kit (MCE, Monmouth Junction, NJ, USA) was used to detect the viability of LUAD cells. H1975 and A549 cells at logarithmic growth phase (1 × 10^3^) were seeded in 96-well plates in triplicate and co-cultured with a conditioned medium (CM). Once the cells adhered, CCK-8 reagents (10 mL/well) were added at the indicated times and incubated for 2 h. Then the optical density of each well was measured at 450 nm using a spectrophotometric plate reader. The measurements were performed once per day for 4 continuous days. For colony formation assay, a total of 1 × 10^3^ H1975 or A549 cells per well were seeded in a 6-well plate and triplicate wells were seeded and co-cultured with CM. After 10 days, cells were stained with a Crystal Violet Staining Solution (C0121, Beyotime).

### Flow cytometry assay

For cell cycle assay, the cells were collected, treated, and fixed overnight with anhydrous ethanol (pre-cooled at −20 °C) at 4 °C. Cells were washed and collected and stained by 400 μL PI Staining Solution/RNase solution for 30–60 min in the dark at 4 °C, and then analyzed by FCM. For apoptosis assay, H1975 or A549 cells were stained with the FITC Annexin V apoptosis detection kit (556547, BD Biosciences) or PE Annexin V apoptosis detection Kit (559763, BD Biosciences) according to the manufacturer protocol and analyzed early-stage and late-stage apoptosis by FACS (FACS AriaTM IIII, BD Biosciences). All data were collected using the BD FACSDiva software and analyzed using FlowJo Software. Anti-human CD45, CD66b, CD10, CD11b, and CD62L antibodies were used to detect differences in peripheral blood neutrophil phenotypes between healthy donors and LUAD patients with distant metastases.

### Cell invasion assay

Transwell assay was performed to ascertain cell invasion using Transwell apparatus (Corning Life Sciences, Corning, NY, USA) with diluted matrigel (BD Bioscience, CA). H1975 or A549 cells (2 × 10^5^) in 200 μL serum-free medium were seeded into the upper chamber. CM of different groups was added to the upper chamber, with or without Alvelestat (10 μM, HY-15651, MCE). After incubation at 37 °C for 24 h, the cells were fixed with 4% paraformaldehyde, stained by crystal violet, and then photographed under a microscope.

### Reverse transcription-quantitative PCR

Neutrophils were isolated from peripheral blood of healthy volunteers and advanced lung cancer patients or mice, or lung cancer cells were co-cultured with neutrophils for 12 h to extract neutrophils and lung cancer cells respectively, and then total RNA from neutrophils or lung cancer cells was extracted with TRIzol reagent (Thermo, 15596026) and genomic DNA was removed using DNA-freeTM Kit DNase Treatment and Removal Reagents (Thermo, AM1906) according to the manufacturer’s protocol. Total RNA was then converted to cDNA using the High-Capacity cDNA Reverse Transcription Kit (Thermo, 4368814). Quantitative real-time PCR reactions were performed according to the protocol of the iTaq Univer SYBR Green Supermix (Bio-Rad,1725125). The sequence of primers used for mRNA are listed in Supplementary Table 4. ΔΔCt method was used for normalized expression. Data analysis was performed in accordance with MIQE guidelines.

### Lentiviral transduction

The full-length TPM2 cDNA-overexpressing lentiviral construct and the empty vector, provided by Genechem (Shanghai, China), were transfected into A549 and H1975 cells. Suppression of TPM2 expression was performed by shRNA interference. TPM2-shRNA recombinant lentiviruses and the negative control (vector) were transfected into HCC827 cells. Uninfected cells were used for empty control (control). After being selected by puromycin, TPM2 overexpression or knockdown in stable cells was verified by Western blotting.

### Western blot assay

Expression of the indicated molecules was determined using the western blotting assay. The following antibodies were used: rabbit anti-ELANE antibody (Abcam, Cambridge, UK), rabbit anti-CTSG antibody, rabbit anti-PR3antibody and rabbit anti-granzyme A antibody, rabbit anti-TPM2 antibody (Abcam, Cambridge, UK), rabbit anti-p21 antibody (Abcam, Cambridge, UK), rabbit anti-p53 antibody (Abcam, Cambridge, UK), rabbit anti-E-cad antibody, rabbit anti-Vim antibody, rabbit Snail antibody, rabbit ZEB1 antibody, rabbit Bax antibody, rabbit Caspase 3 antibody, rabbit Caspase 9 antibody, rabbit Cyclin D1 antibody, rabbit Cyclin E1 antibody, rabbit anti-p38/MAPK antibody, rabbit anti-phosphorylated p38/MAPK (anti-p-p38/MAPK) antibody, rabbit anti-ERK1/2 antibody, rabbit anti-p-ERK1/2 antibody, rabbit anti-NF-kB p65 antibody, rabbit anti-p-NF-kB p65 antibody, rabbit anti-PI3 Kinase p85 antibody, rabbit anti-p-PI3 Kinase p85 antibody, rabbit anti-AKT antibody, rabbit anti-p-AKT antibody, rabbit anti-JNK antibody, rabbit anti-p-JNK antibody, (Cell Signaling Technology, MA, USA). rabbit anti-YPA antibody, rabbit anti-p-YAP antibody (Abcam, Cambridge, UK). Relative levels were quantified and normalized with GAPDH in the same sample with density analysis.

### Enzyme-linked immunosorbent assay (ELISA)

Cytokines were detected in human or mouse neutrophil culture medium according to the manufacturer’s instructions. ELISA kits were used to assay the levels of human IL1β(88-7621-88, ThermoFisher), human IL8 (431507, Biolegend), human IL5 (430404, Biolegend), human IL6 (430507, Biolegend), human TNFɑ (430207, Biolegend), human GM-CSF (432007, Biolegend), mouse IL6 (88-7064-88, ThermoFisher), mouse IL8 (BLL-S7365G, Baililaibo). Then, signals were detected using a TMB solution and read at 450 nm.

### Two-chamber neutrophil migration assays

Briefly, 5 × 10^5^ freshly isolated neutrophils in RPMI 1640 were added to the upper chamber (363096, BD), and CM-A-neu or CM-H-neu was added to the lower chamber as the chemoattractant. The migrated cells in the lower chamber were counted after 3 h.

### RNA sequencing

Total RNA was isolated and reversely transcribed into cDNA to generate an indexed Illumina library, followed by sequencing at the Beijing Genomics Institute (Beijing, China) using a BGISEQ-500 platform. Significant differential expression of a gene was defined as a >2-fold expression difference vs the control with an adjusted *p* value less than 0.05. A heat map was analyzed by Gene Ontology (GO) using Cluster software and visualized with Java Treeview. DEGs were analyzed by GO using the AMIGO and DAVID software. The enrichment degrees of DEGs were analyzed using KEGG annotations.

### TdT-mediated dUTP nick-end labeling (TUNEL) assay

Mouse tumor paraffin-embedded tissues were stained using the TUNEL assay kit for apoptosis (Roche Diagnostics GmbH, Mannheim, Germany) according to the manufacturer’s instructions. Use an optical microscope to observe and photograph.

### Mouse models

Male C57BL/6 mice (Vitalriver, Nanjing, China) aged 6–8 weeks were used in all animal experiments. An Institutional Animal Care and Use Committee (IACUC) of Yunnan University has approved these studies and all mouse experiments were performed according to the ethics permission (YNU20210802). For Orthotopic (intralung) tumor model, A total of 1.0 × 10^6^ LLC-luc-GFP cells (LLC with or without Tpm2 overexpression) in a solution containing 20 µL culture medium and 20 µL Matrigel (BD Biosciences, NJ, USA) were directly injected through the intercostal space into the lung to a depth of 3 mm using a 29-G needle permanently attached to a 0.5-mL insulin syringe (Becton Dickinson, NJ, USA). The mice were then allowed to rest on a heating carpet until fully recovered. For Alvelestat treatment, Alvelestat (6 mg/kg) was administered orally twice daily until the mice were euthanized. Tumor burdens were quantified by bioluminescence imaging (BLI) on the IVIS Lumina III (Perkin Elmer) platform, BLI data was analyzed using Living Image software (RRID:SCR_014247); BLI signal was reported as average flux (photons per second/area [mm^2^]). All animals were anesthetized with isoflurane gas. Tumor growth in mice was monitored every week.

### Statistical analyses

Data were presented as the mean ± SD or mean ± SEM and *p* < 0.05 was considered significant. Statistical analysis was performed using GraphPad Prism 8 software. Pearson’s correlation analyses were used to calculate the regression and correlation between the two groups. As indicated in the Figure legends, all assays were performed in three biological replicates unless stated otherwise. Representative micrographs and western blot shown in the Figures were repeated three times independently with similar results.

## Supplementary information

Original western blots

Supplementary Figure legend

Supplementary Figure 1

Supplementary Figure 2

Supplementary Figure 3

Supplementary Figure 4

Supplementary Table 1

Supplementary Table 2

Supplementary Table 3

Supplementary Table 4

## Data Availability

Datasets generated or analyzed during the current study are available from the corresponding author on reasonable request.

## Code availability

The version and parameters for the RNA sequencing analysis package used in this study are available in the Material and Methods section. Any additional information required to reanalyze the data reported in this study is available from the corresponding author on reasonable request.
