# Supplementary material for: Downregulation of tropomyosin 2 promotes the progression of lung adenocarcinoma by regulating neutrophil infiltration through neutrophil elastase
Source: Cell Death Dis. 2025 Apr 8;16(1):264. doi: 10.1038/s41419-025-07531-1 (PMC11978998; doi:10.1038/s41419-025-07531-1)

Original Figure 2 I -A549

Original Figure 2 I -A549 p21：

Figure 2I

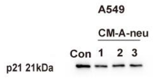

Original Figure 2 I -A549 cyclinA1：

Figure 2I

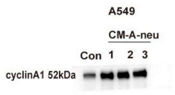

Original Figure 2 I -A549 cyclinB1：

Figure 2I

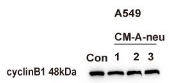

Original Figure 2 I -A549 CDK1：

Figure 2I

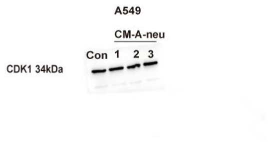

Original Figure 2 I -A549 pro-caspase3：

Figure 2I

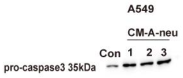

Original Figure 2 I -A549 cleaved-caspase3:

Figure 2I

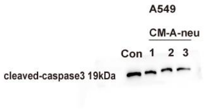

Original Figure 2 I -A549 pro-caspase9:

Figure 2I

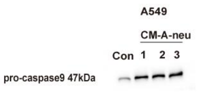

Original Figure 2 I -A549 cleaved-caspase9:

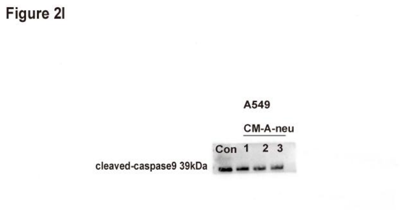

Original Figure 2 I -A549 Bax：

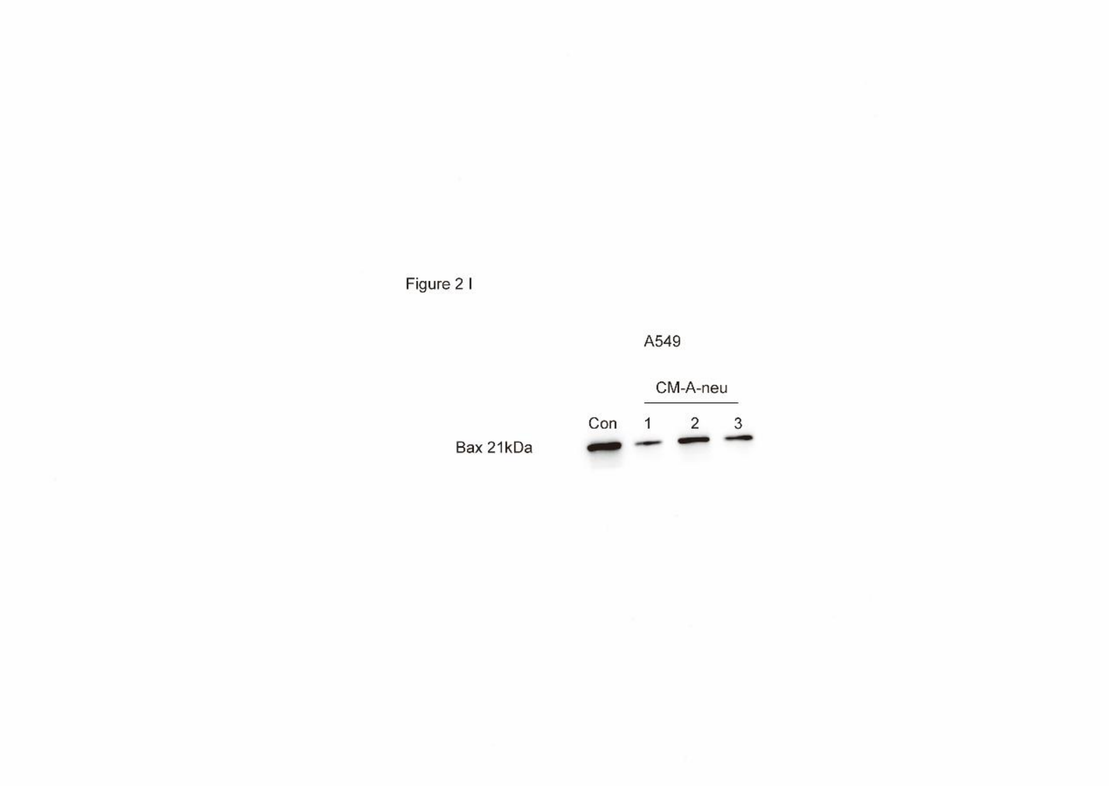

Original Figure 2 I A549 CyclinD1

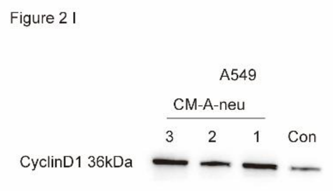

Original Figure 2 I A549 CyclinE1

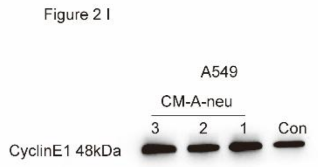

Original Figure 2 I A549 E-cadherin

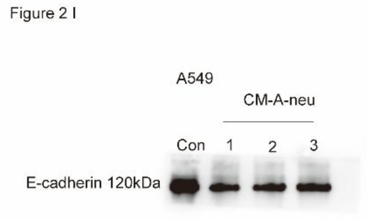

Original Figure 2 I A549 GAPDH

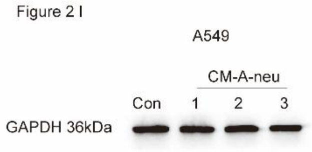

Original Figure 2 I A549 Snail

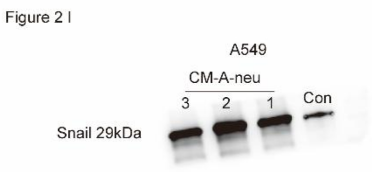

Original Figure 2 I A549 Vimentin

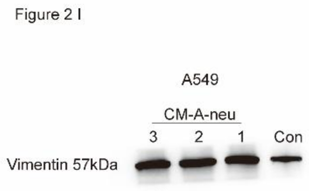

Original Figure 2 I A549 ZEB1

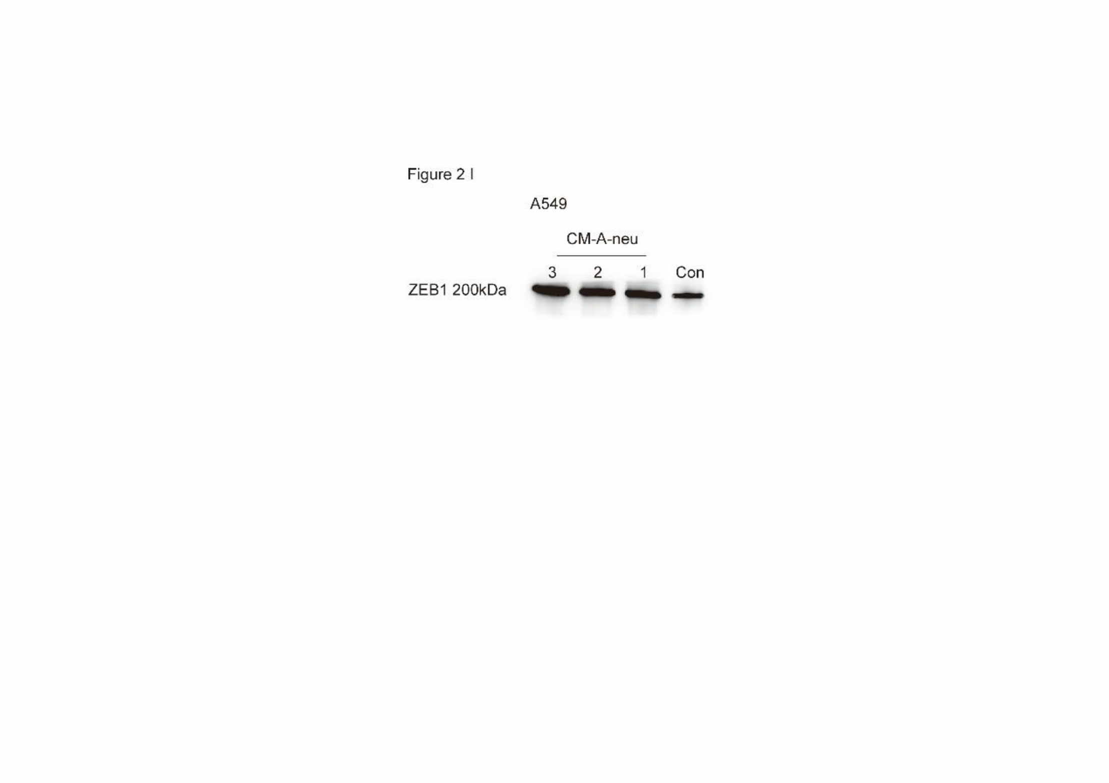

Original Figure 2 I H1975

Original Figure 2 I H1975 p21:

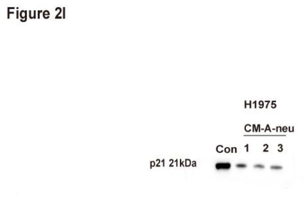

Original Figure 2 I H1975 cyclinA1:

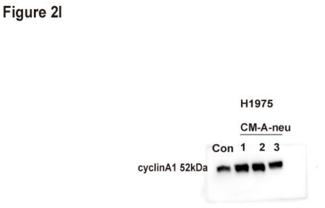

Original Figure 2 I H1975 cyclinB1:

Figure 2I

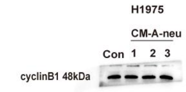

Original Figure 2 I H1975 CDK1:

Figure 2I

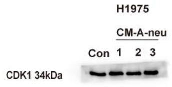

Original Figure 2 I H1975 pro-caspase3:

Figure 2I

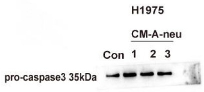

Original Figure 2 I H1975 cleaved-caspase3:

Figure 2I

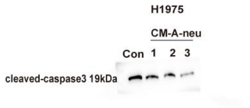

Original Figure 2 I H1975 pro-caspase9:

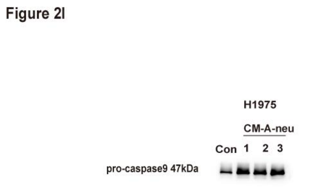

Original Figure 2 I H1975 cleaved-caspase3:

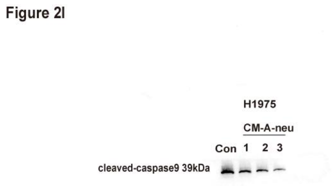

Original Figure 2 I H1975 Bax:

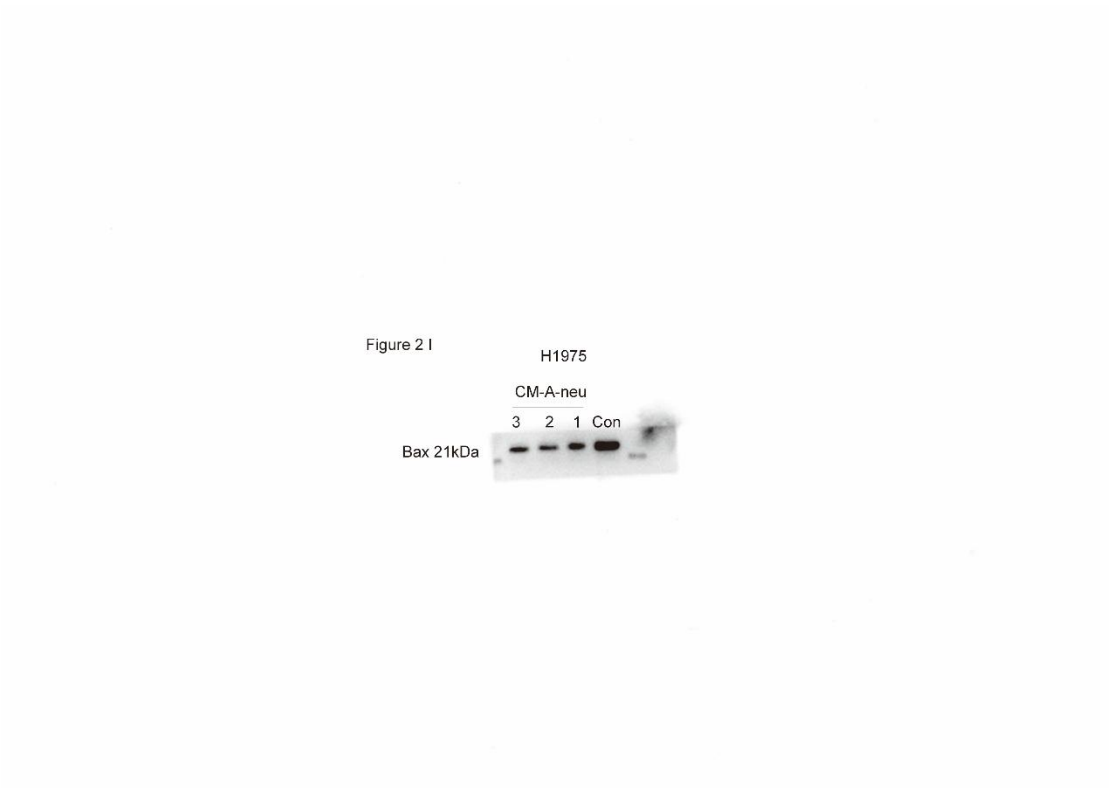

Original Figure 2 I H1975 CyclinD1

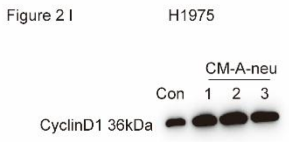

Original Figure 2 I H1975 CyclinE1

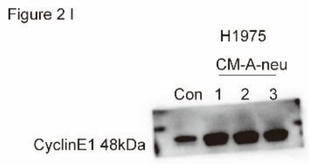

Original Figure 2 I H1975 E-cadherin

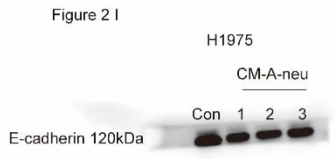

Original Figure 2 I H1975 GAPDH

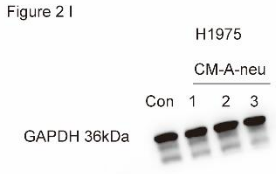

Original Figure 2 I H1975 Snail

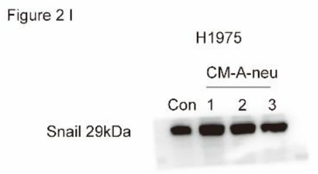

Original Figure 2 I H1975 Vimentin

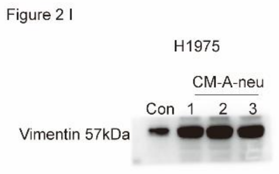

Original Figure 2 I H1975 ZEB1

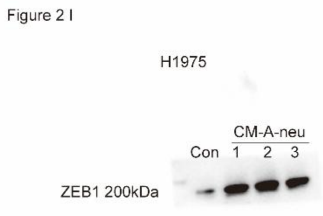

Original Figure 3

Original Figure 3B ELANE

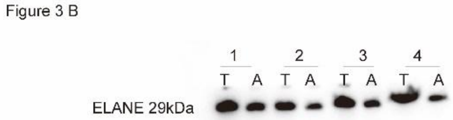

Original Figure 3B ELANE

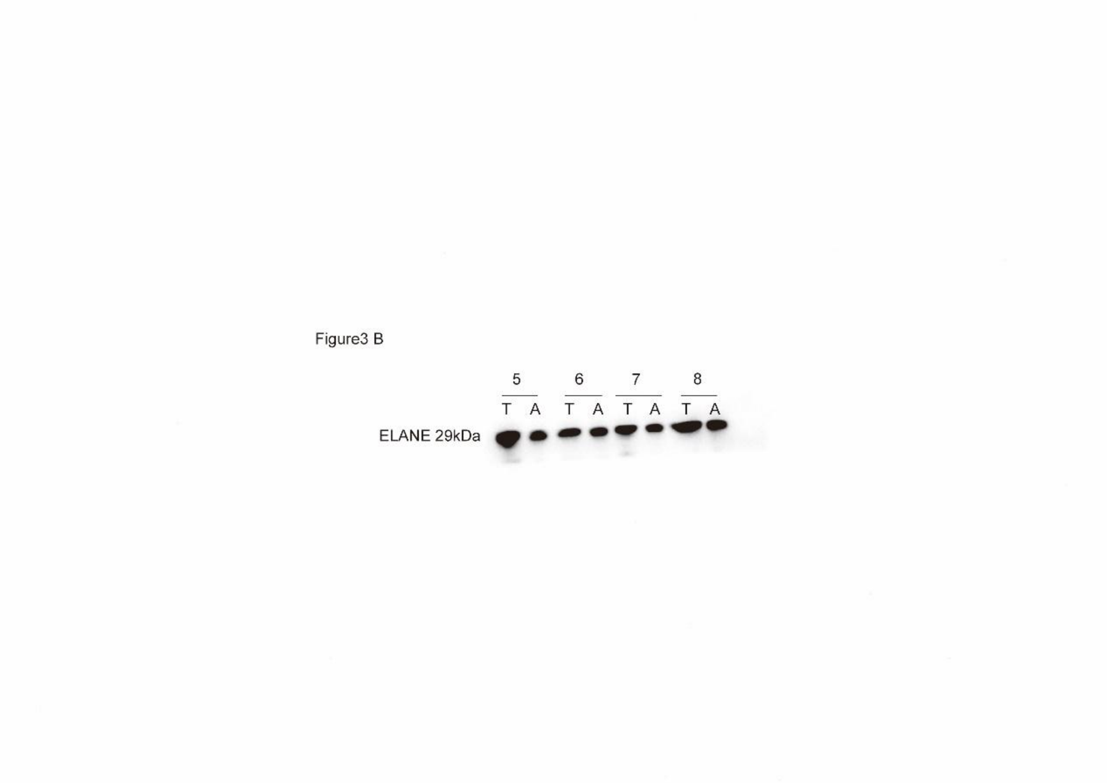

Original Figure 3B ELANE

Figure 3B

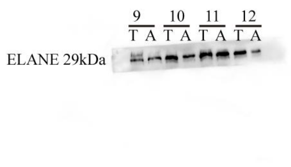

Original Figure 3B GAPDH

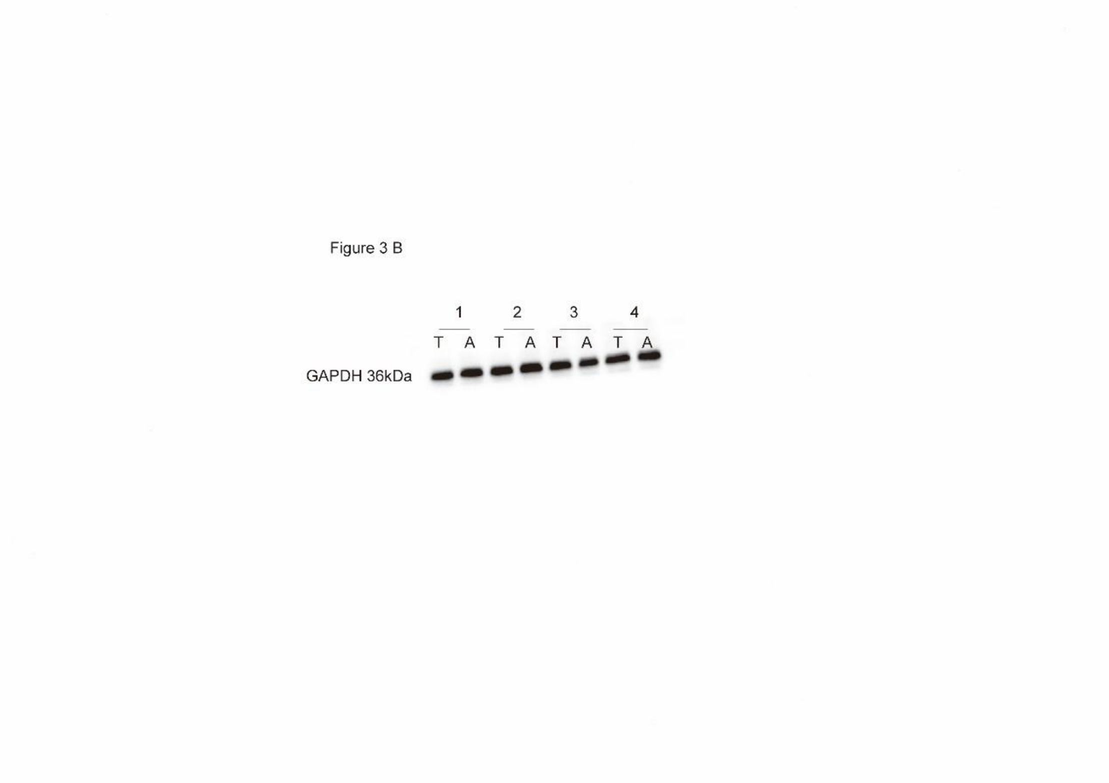

Original Figure 3B GAPDH

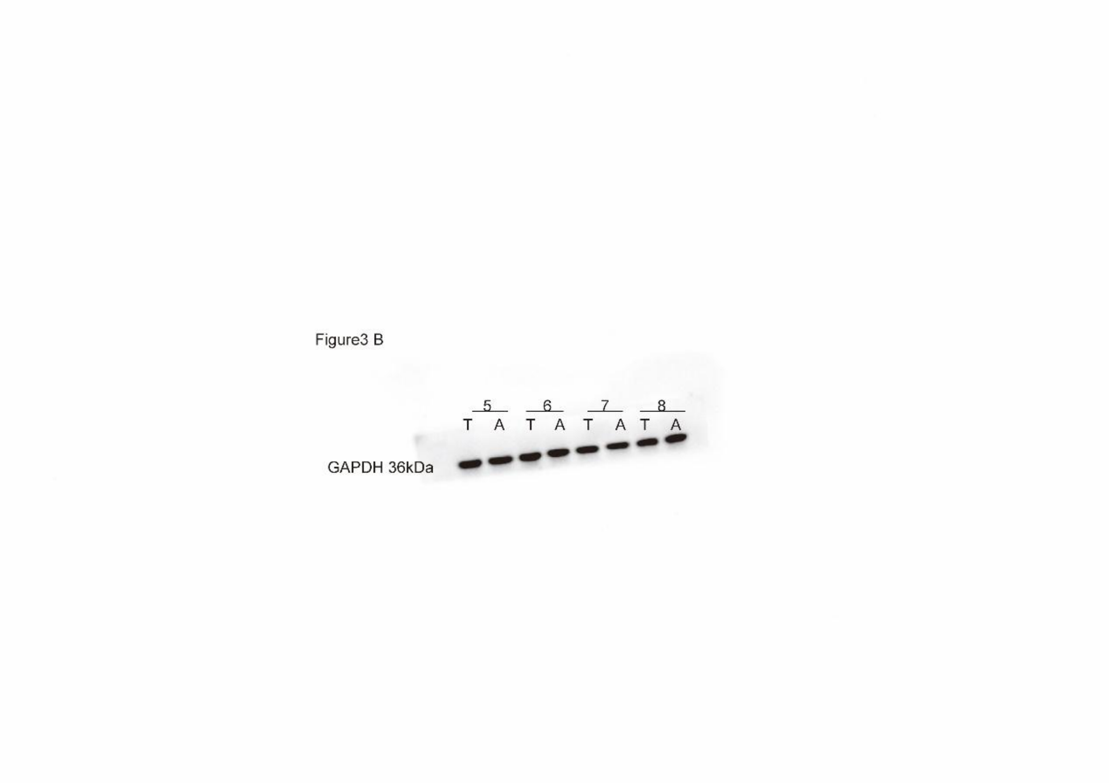

Original Figure 3B GAPDH

Figure 3B

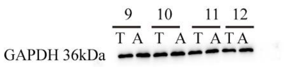

Original Figure 4

Original Figure 4A-A549

Original Figure 4A-A549 p21:

Figure 4A

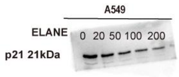

Original Figure 4A-A549 cyclinA1:

Figure 4A

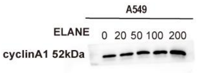

Original Figure 4A-A549 cyclinB1:

Figure 4A

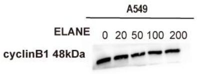

Original Figure 4A-A549 CDK1:

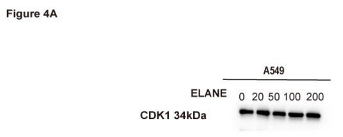

Original Figure 4A-A549 pro-caspase3:

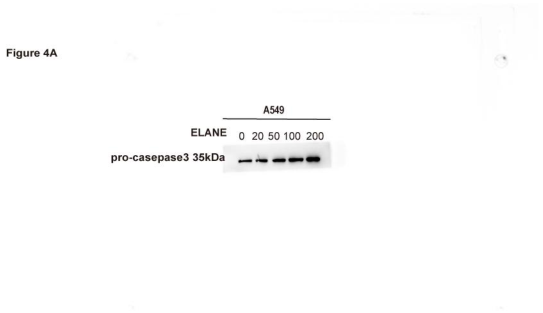

Original Figure 4A-A549 cleaved-caspase3:

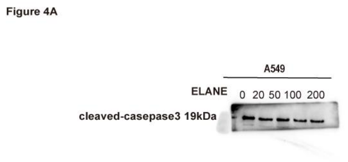

Original Figure 4A-A549 pro-caspase9:

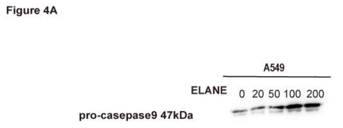

Original Figure 4A-A549 cleaved-caspase3:

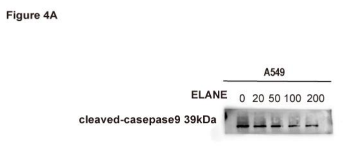

Original Figure 4A-A549 Bax:

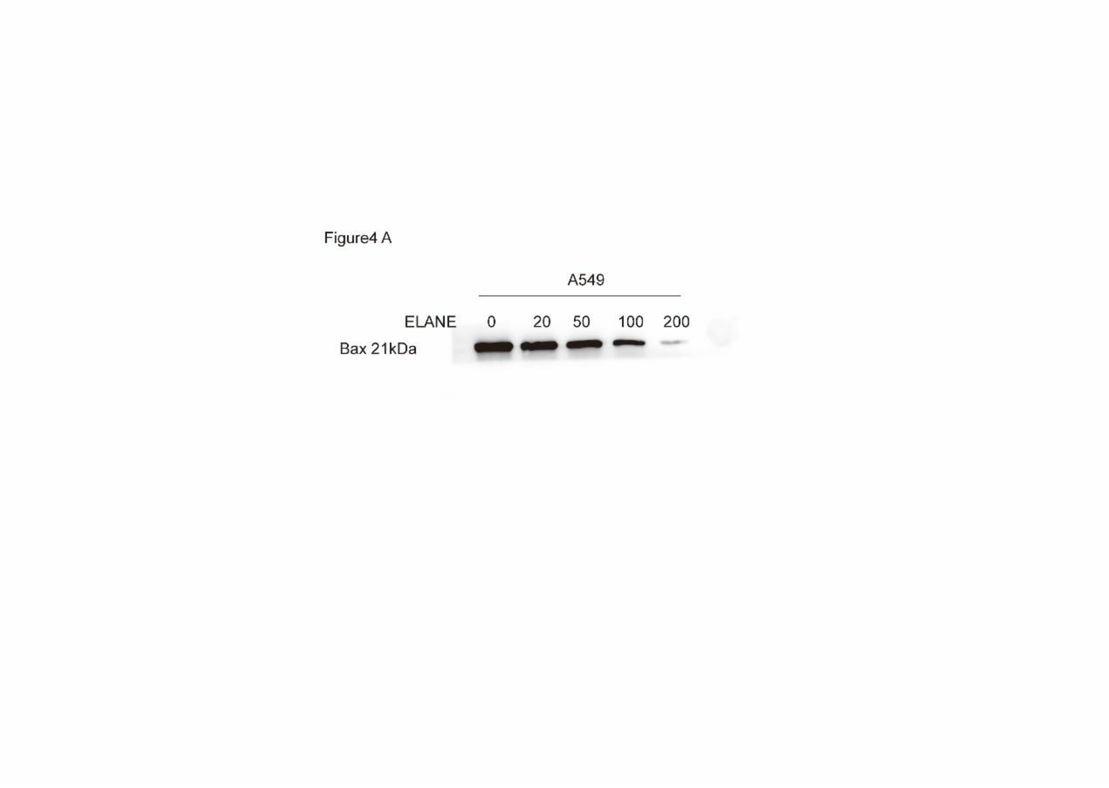

Original Figure 4A -A549 CyclinD1

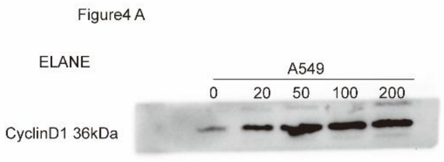

Original Figure 4A -A549 CyclinE1

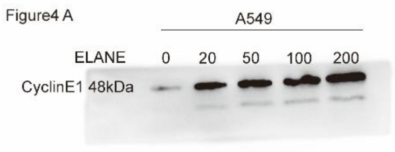

Original Figure 4A -A549 E-cadherin

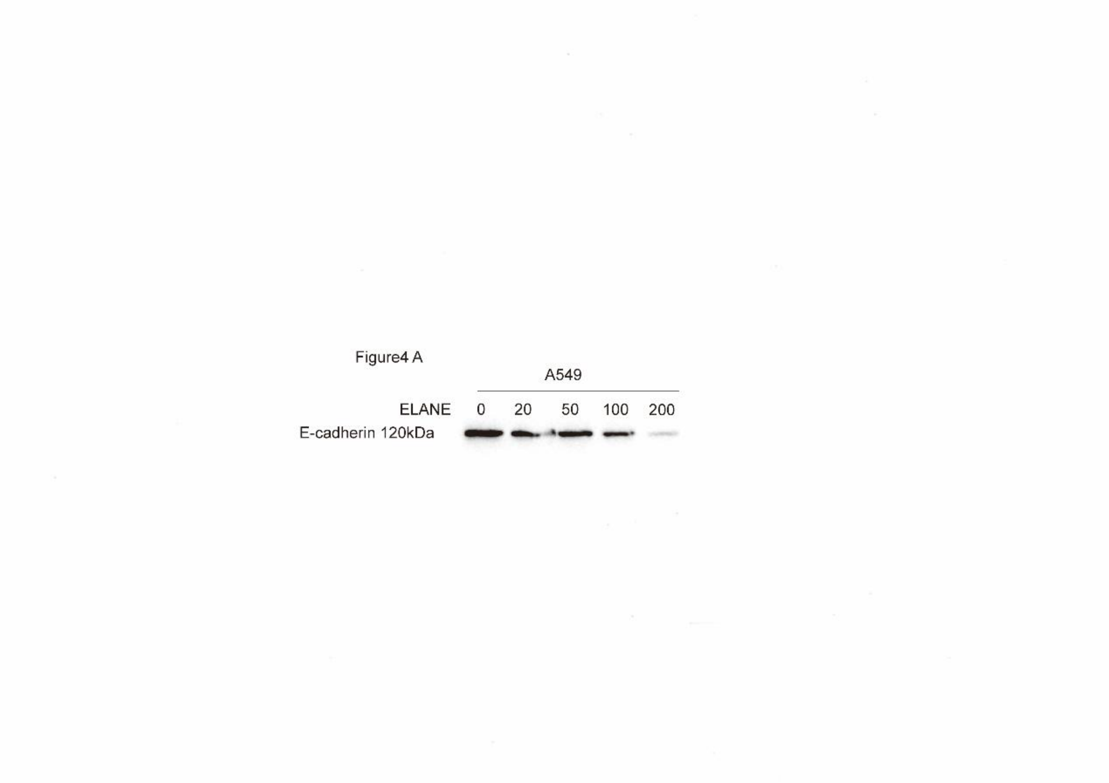

Original Figure 4A -A549 GAPDH

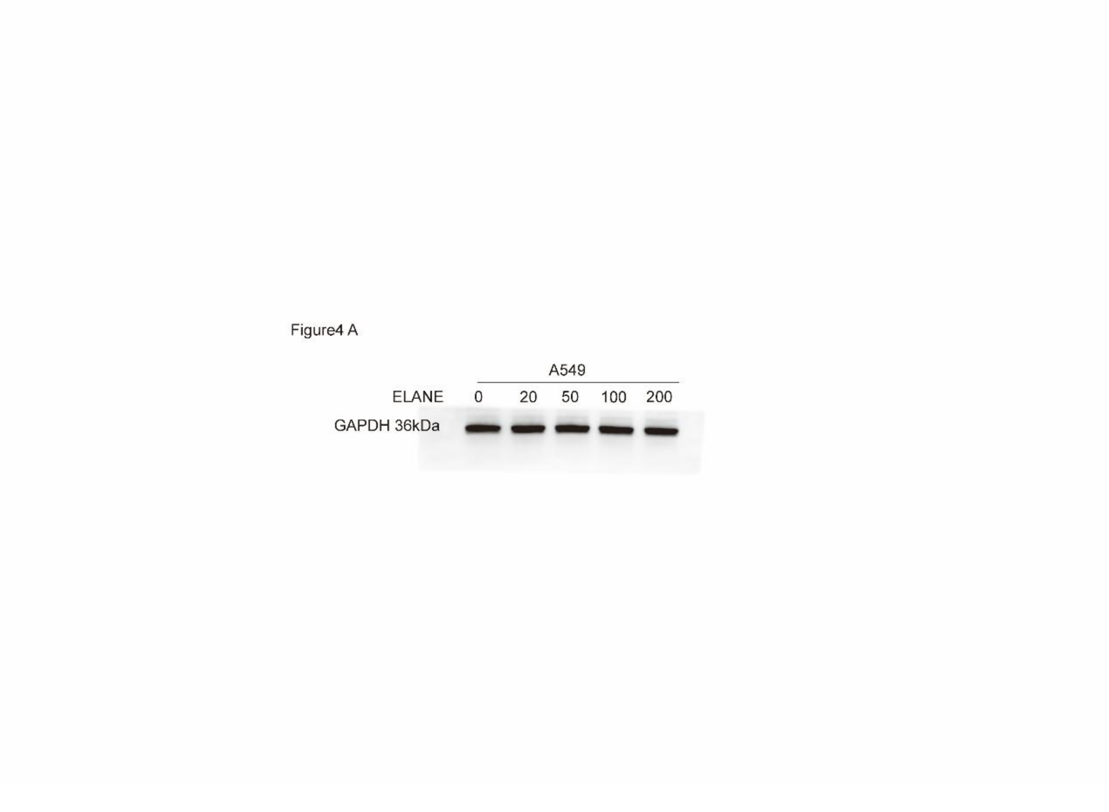

Original Figure 4A-A549 Snail

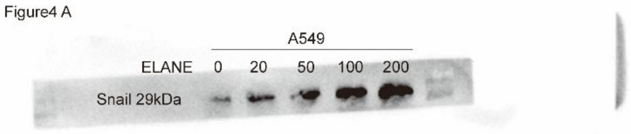

Original Figure 4A-A549 Vimentin

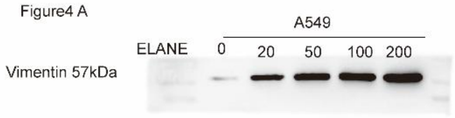

Original Figure 4A -A549 ZEBI

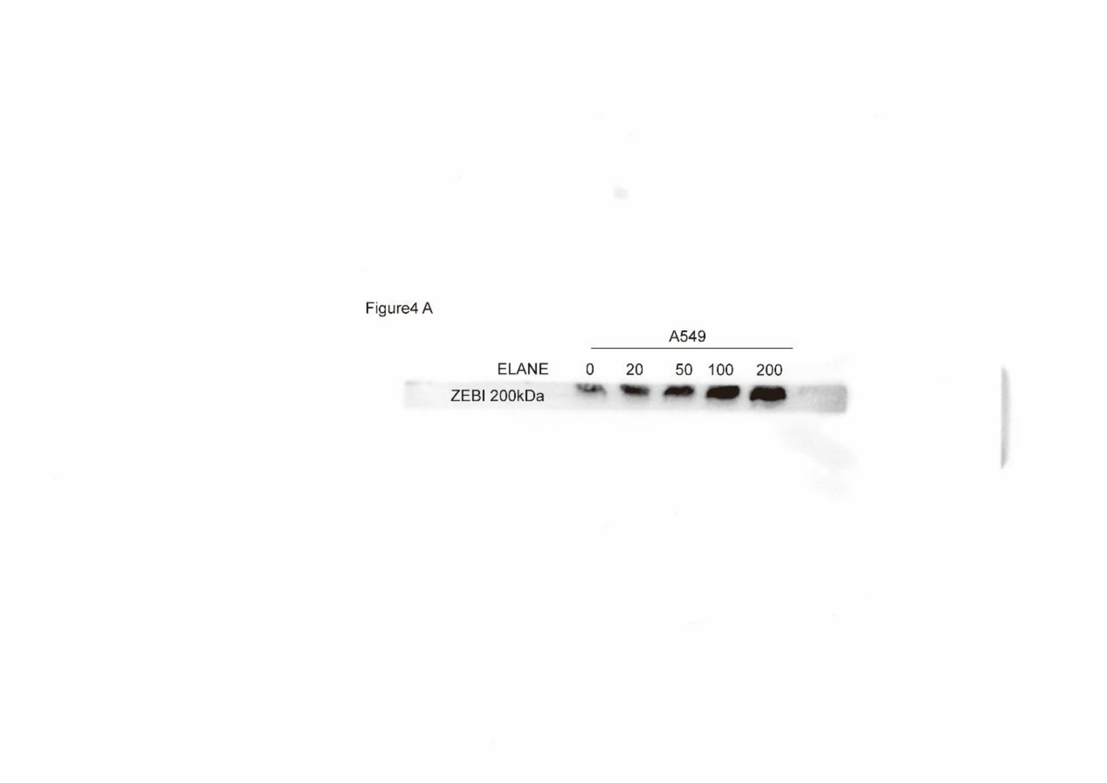

Original Figure 4A -H1975

Original Figure 4A-H1975 p21:

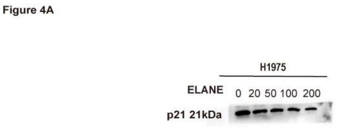

Original Figure 4A-H1975 cyclinA1:

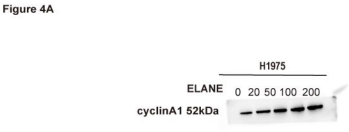

Original Figure 4A-H1975 cyclinB1:

Figure 4A

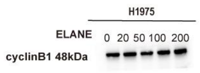

Original Figure 4A-H1975 CDK1:

Figure 4A

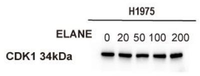

Original Figure 4A-H1975 pro-caspase3:

Figure 4A

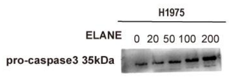

Original Figure 4A-H1975 cleaved-caspase3:

Figure 4A

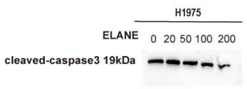

Original Figure 4A-H1975 pro-caspase9:

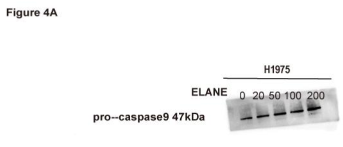

Original Figure 4A-H1975 cleaved-caspase9:

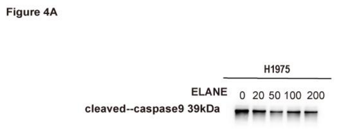

Original Figure 4A-H1975 Bax:

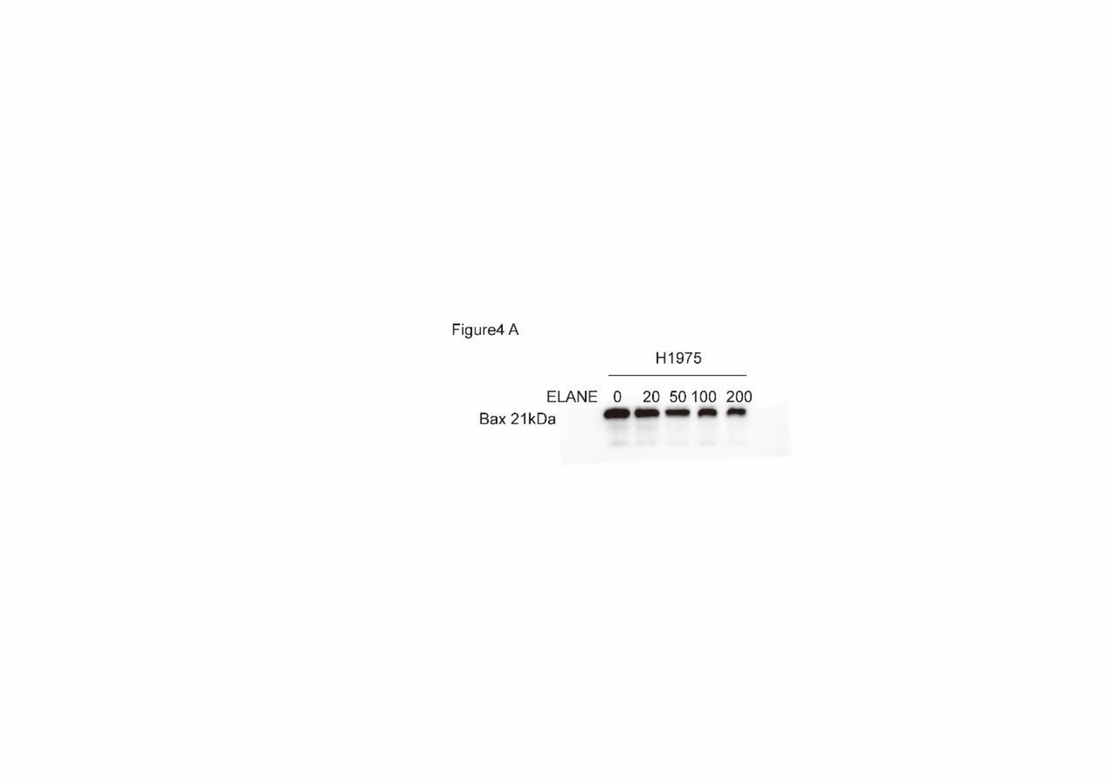

Original Figure 4A- H1975 CyclinD1

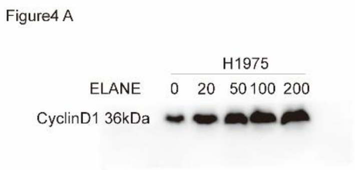

Original Figure 4A- H1975 CyclinE1

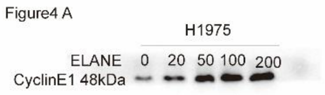

Original Figure 4A- H1975 E-cadherin

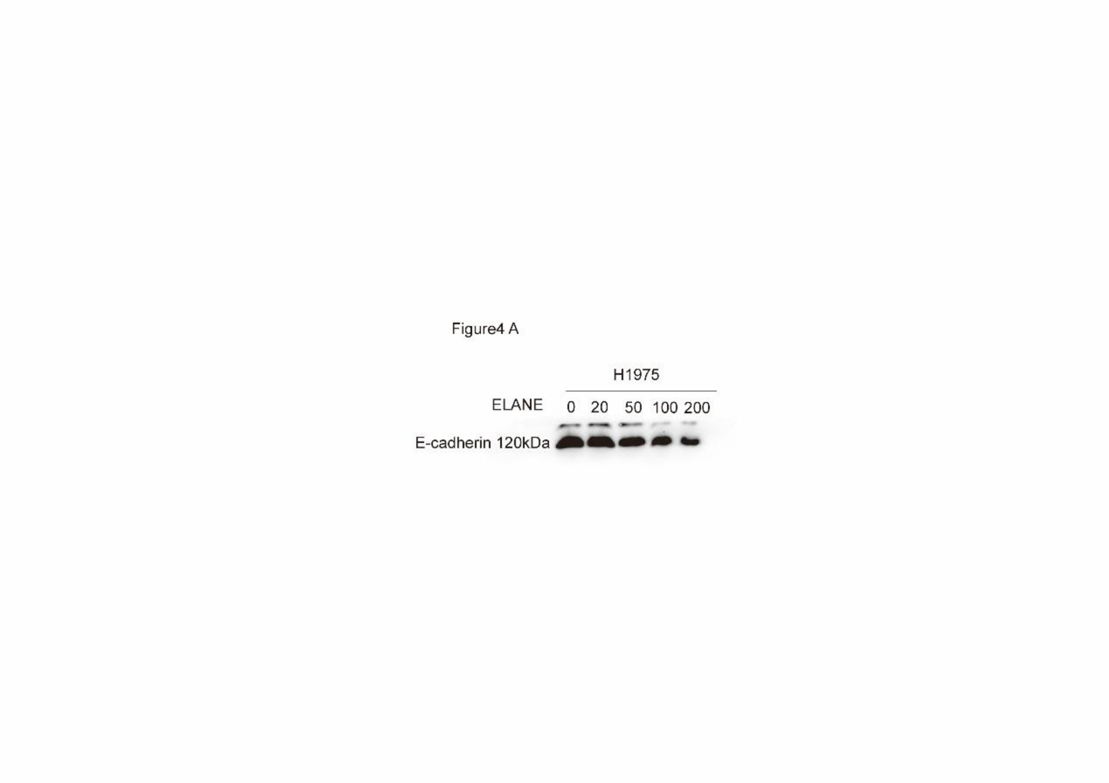

Original Figure 4A- H1975 GAPDH

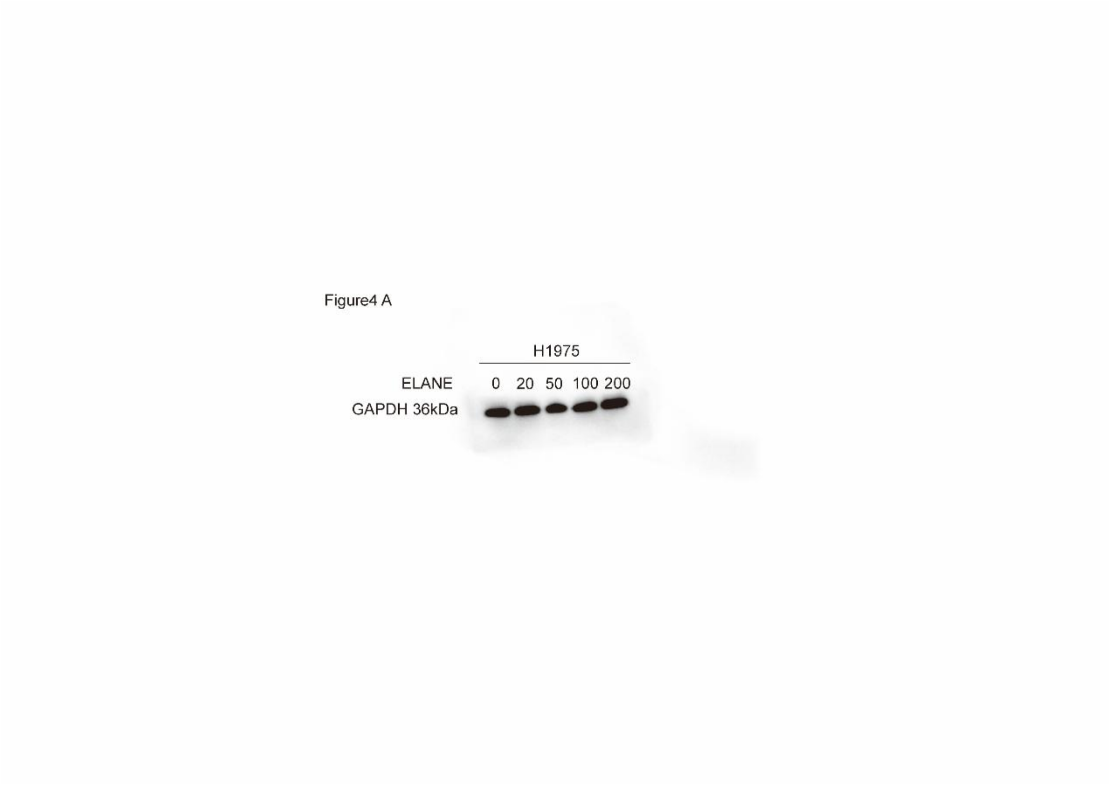

Original Figure 4A- H1975 Snail

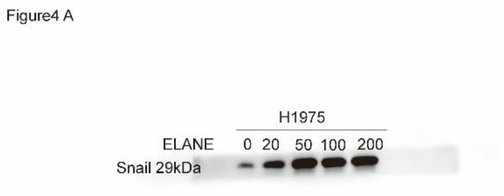

Original Figure 4A- H1975 Vimentin

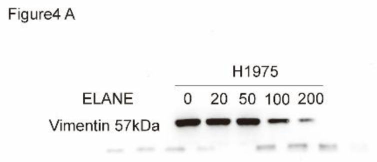

Original Figure 4A- H1975 ZEBI

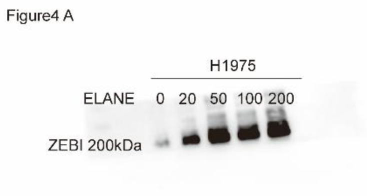

Original Figure 4D

Original Figure 4D cyclinD1:

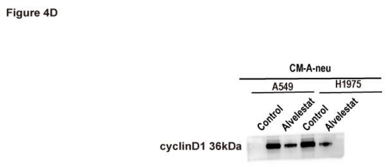

Original Figure 4D cyclinE1:

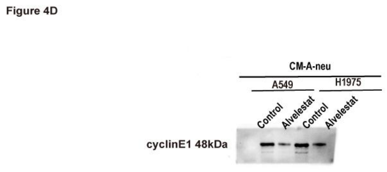

Original Figure 4D cyclinA1:

Figure 4D

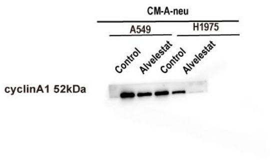

Original Figure 4D cyclinB1:

Figure 4D

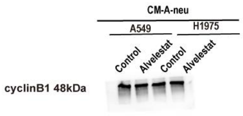

Original Figure 4D CDK1:

Figure 4D

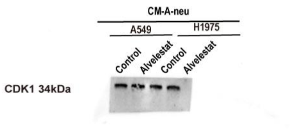

Original Figure 4D GAPDH:

Figure 4D

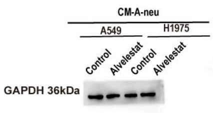

Original Figure 4J A549

Original Figure 4J -A549 CTGF:

Figure 4J

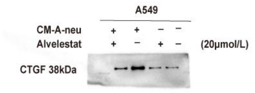

Original Figure 4J -A549 CYR61:

Figure 4J

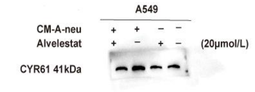

Original Figure 4J -A549 cyclinA1:

Figure 4J

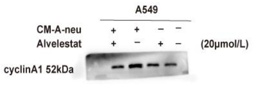

Original Figure 4J -A549 cyclinB1:

Figure 4J

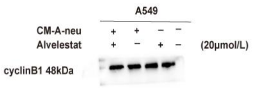

Original Figure 4J -A549 CDK1:

Figure 4J

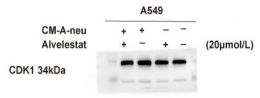

Original Figure 4J -A549 pro-caspase3:

Figure 4J

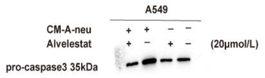

Original Figure 4J -A549 cleaved-caspase3:

Figure 4J

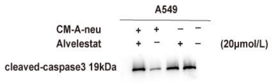

Original Figure 4J -A549 pro-caspase9:

Figure 4J

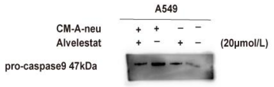

Original Figure 4J -A549 cleaved-caspase9:

Figure 4J

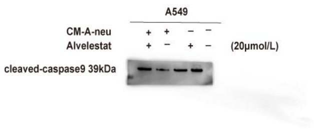

Original Figure 4J -A549 pro-PARP:

Figure 4J

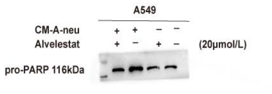

Original Figure 4J -A549 cleaved-PARP:

Figure 4J

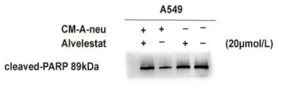

Original Figure 4J -A549 Bax:

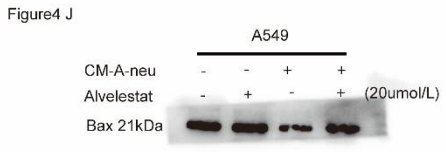

Original Figure 4J- A549 CyclinD1

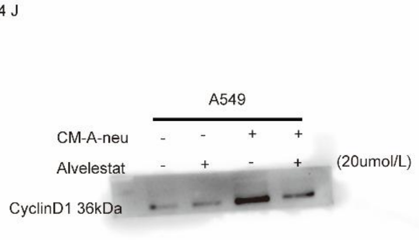

Original Figure 4J -A549 E-cadherin

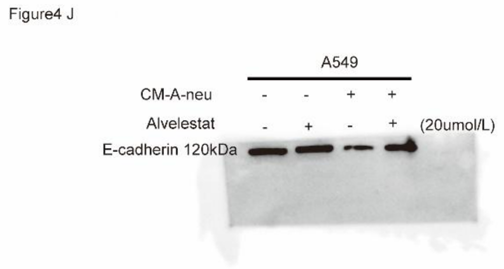

Original Figure 4J -A549 GAPDH

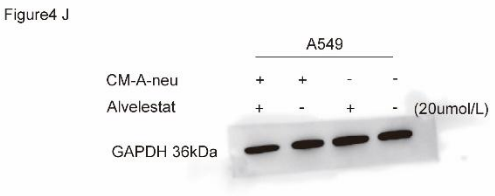

Original Figure 4J- A549 p-YAP:

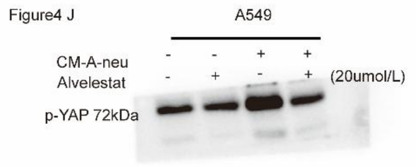

Original Figure 4J- A549 Vimentin

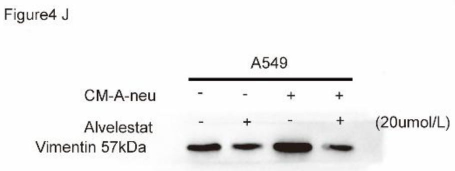

Original Figure 4J- A549 YAP

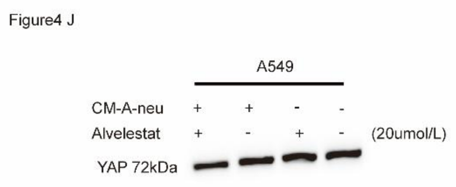

Original Figure 5

Original Figure 5E- A-neu

Original Figure 5E -A-neu ELANE

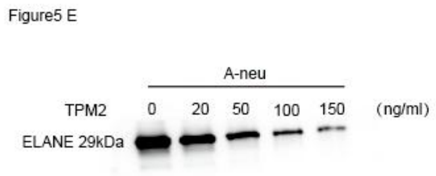

Original Figure 5E- A-neu GAPDH

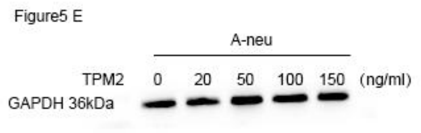

Original Figure 5E- A-neu p38MAPK

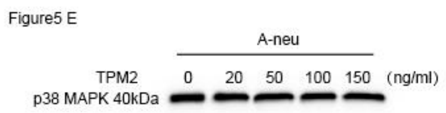

Original Figure 5E- A-neu p-p38MAPK

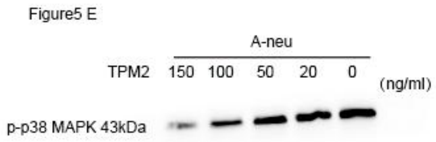

Original Figure 5F- A-neu

Original Figure 5F -A-neu ELANE (A549-TPM2)

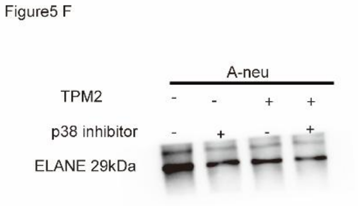

Original Figure 5F -A-neu GAPDH(A549-TPM2)

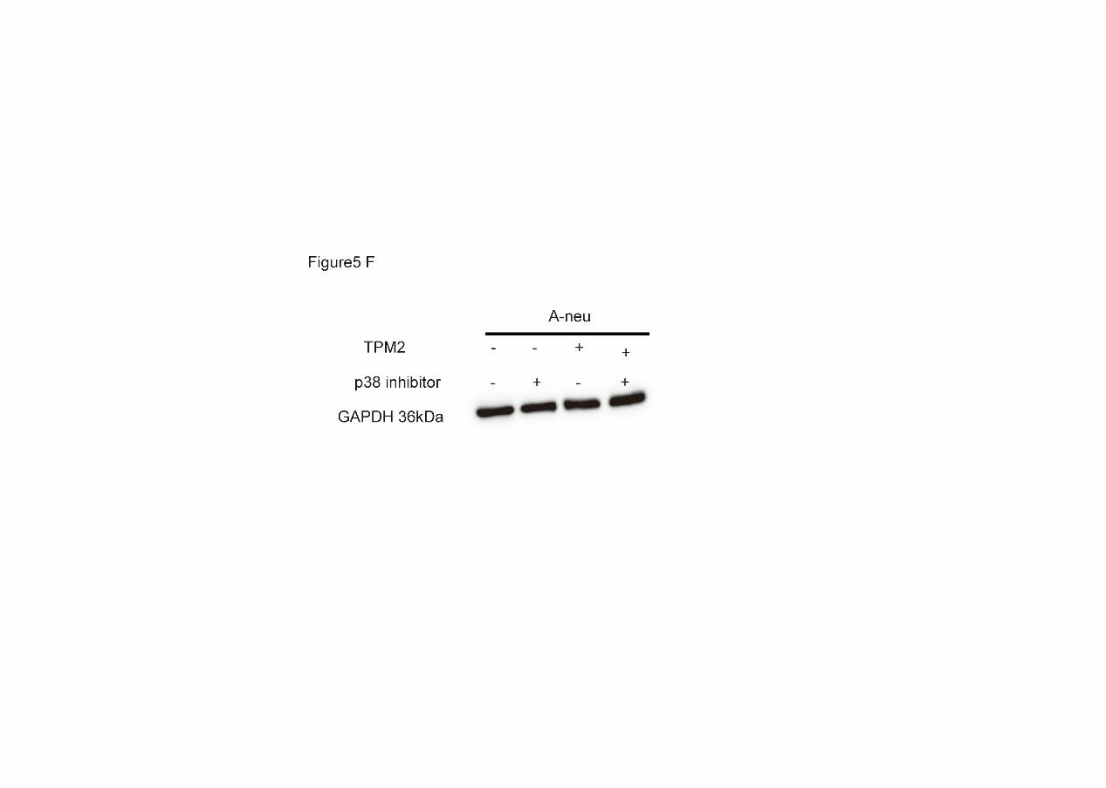

Original Figure 5G- A-neu

Original Figure 5G -A-neu ELANE (HCC827-sh-TPM2)

Figure 5G

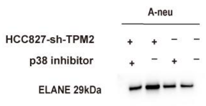

Original Figure 5G -A-neu GAPDH (HCC827-sh-TPM2)

Figure 5G

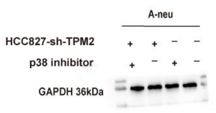

Original Figure 5H -A549

Original Figure 5H -A549 YAP:

Figure 5H

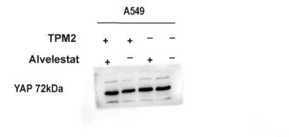

Original Figure 5H -A549 p-YAP:

Figure 5H

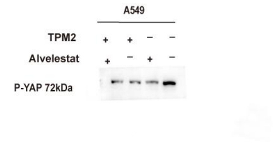

Original Figure 5H -A549 CTGF:

Figure 5H

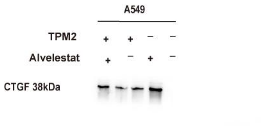

Original Figure 5H -A549 CYR61:

Figure 5H

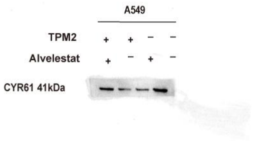

Original Figure 5H -A549 cyclinA1:

Figure 5H

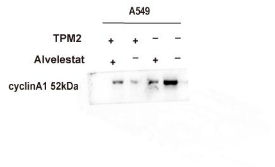

Original Figure 5H -A549 cyclinB1:

Figure 5H

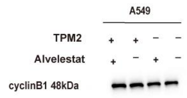

Original Figure 5H -A549 CDK1:

Figure 5H

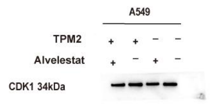

Original Figure 5H -A549 pro-caspase3:

Figure 5H

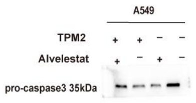

Original Figure 5H -A549 cleaved-caspase3:

Figure 5H

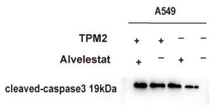

Original Figure 5G- A549 pro-caspase9:

Figure 5H

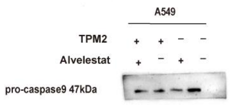

Original Figure 5H -A549 cleaved-caspase9:

Figure 5H

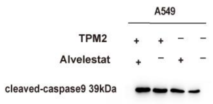

Original Figure 5H -A549 pro-PARP:

Figure 5H

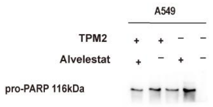

Original Figure 5H -A549 cleaved-PARP:

Figure 5H

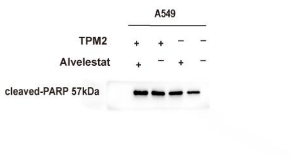

Original Figure 5G -A549 CyclinD1

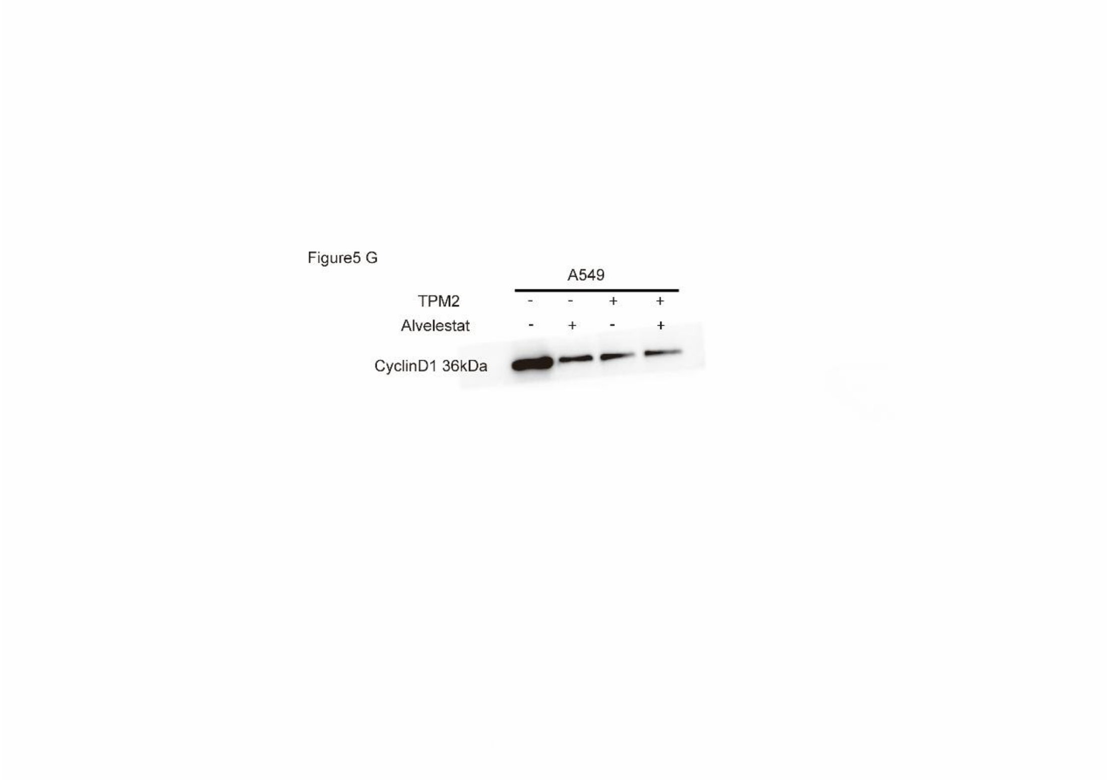

Original Figure 5G -A549 CyclinE1

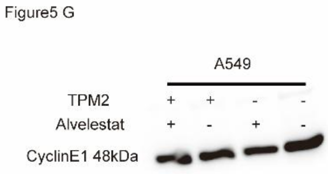

Original Figure 5G -A549 Bax

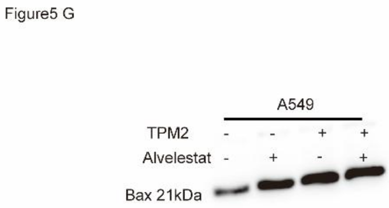

Original Figure 5G -A549 E-cadherin

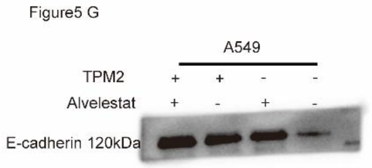

Original Figure 5G -A549 GAPDH-1

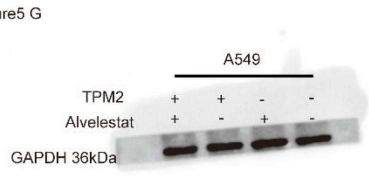

Original Figure 5G -A549 GAPDH-2

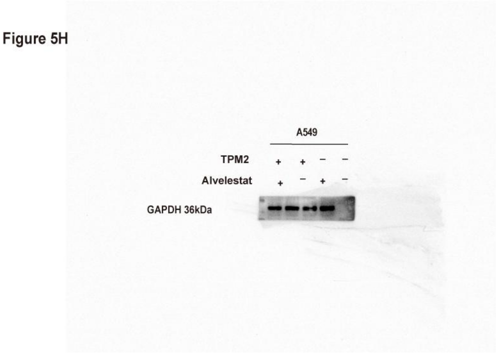

Original Figure 5G -A549 Vimentin

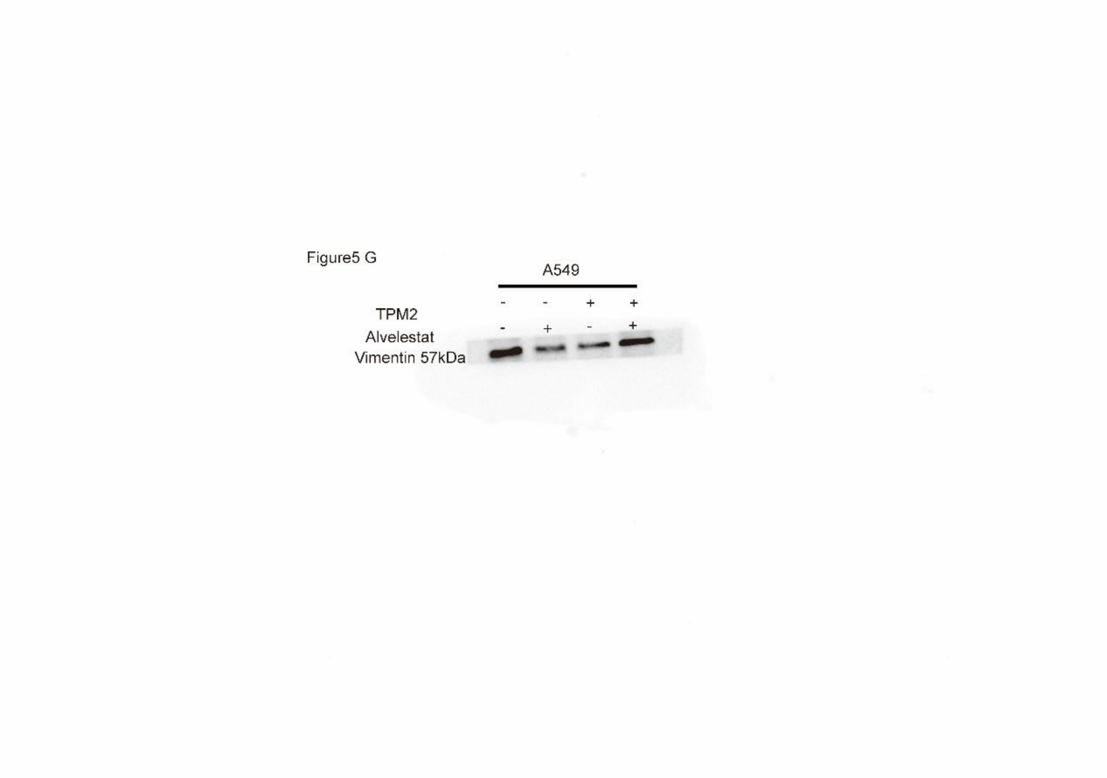

Original Figure 5I -HCC827

Original Figure 5I -HCC827 YAP:

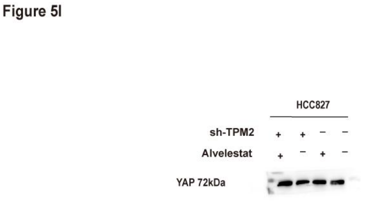

Original Figure 5I -HCC827 p-YAP:

Figure 5I

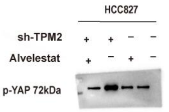

Original Figure 5I -HCC827 CTGF:

Figure 5I

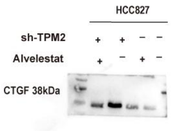

Original Figure 5I -HCC827 CYR61:

Figure 5I

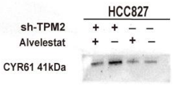

Original Figure 5I -HCC827 cyclinD1:

Figure 5I

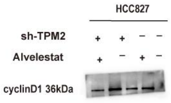

Original Figure 5I -HCC827 cyclinE1:

Figure 5I

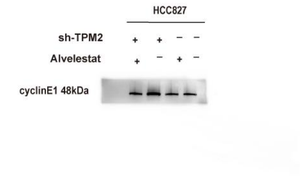

Original Figure 5I -HCC827 cyclinA1:

Figure 5I

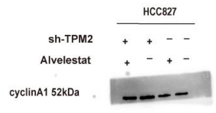

Original Figure 5I -HCC827 cyclinB1:

Figure 5I

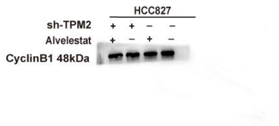

Original Figure 5I -HCC827 CDK1:

Figure 5I

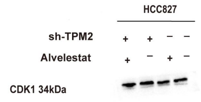

Original Figure 5I -HCC827 GAPDH-1:

Figure 5I

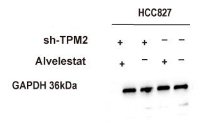

Original Figure 5I -HCC827 Bax:

Figure 5I

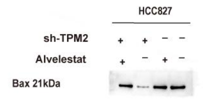

Original Figure 5I -HCC827 pro-caspase3:

Figure 5I

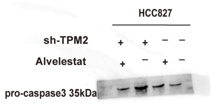

Original Figure 5I -HCC827 cleaved-caspase3:

Figure 5I

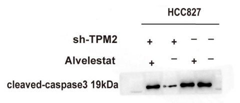

Original Figure 5I -HCC827 pro-caspase9:

Figure 5I

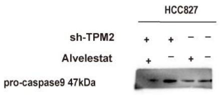

Original Figure 5I -HCC827 cleaved-caspase9:

Figure 5I

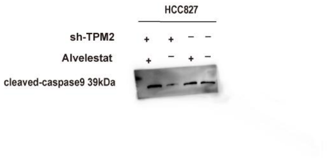

Original Figure 5I -HCC827 pro-PARP:

Figure 5I

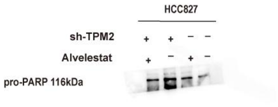

Original Figure 5I -HCC827 cleaved-PARP:

Figure 5I

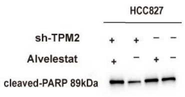

Original Figure 5I -HCC827 E-cadherin:

Figure 5I

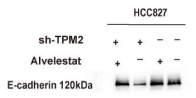

Original Figure 5I -HCC827 Vimentin:

Figure 5I

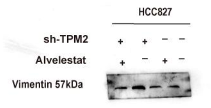

Original Figure 5I -HCC827 GAPDH-2:

Figure 5I

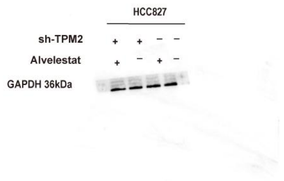

Original Figure 6

Original Figure 6F -A-neu

Original Figure 6F- AKT

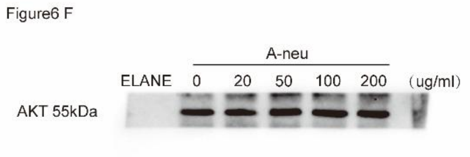

Original Figure 6F -ERK1/2

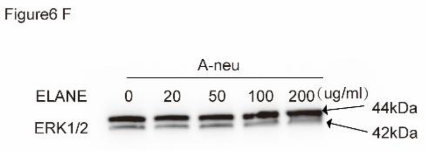

Original Figure 6F- GAPDH

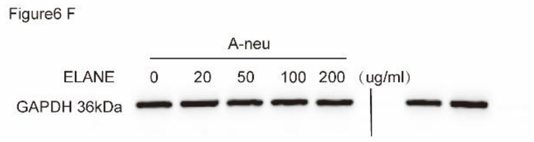

Original Figure 6F- NF-κB

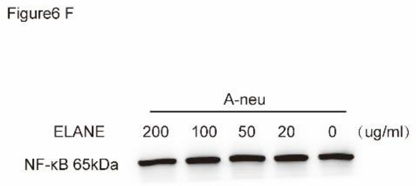

Original Figure 6F -p-AKT

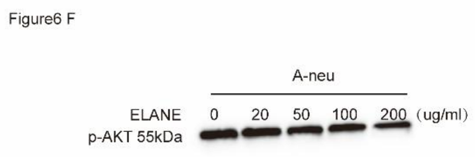

Original Figure 6F- p-ERK1_2

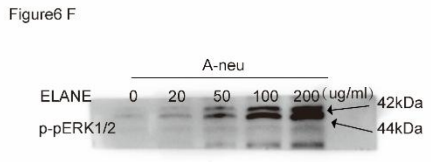

Original Figure 6F- PI3K

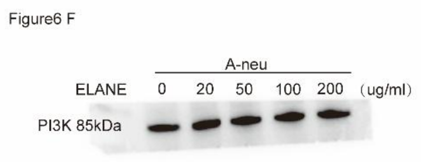

Original Figure 6F -p-NF-κB

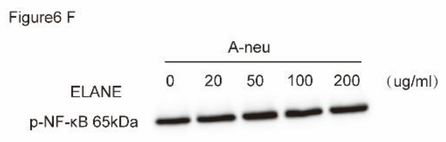

Original Figure 6F -p-PI3K

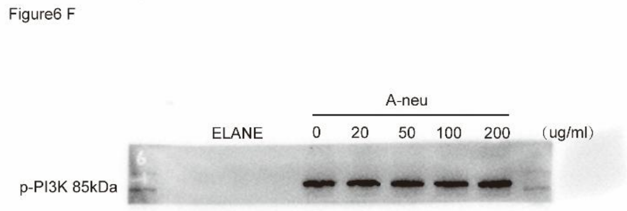

Original Figure 6G -A-neu
Original Figure 6G- A-neu GAPDH

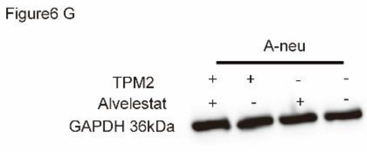

Original Figure 6G- ERK1_2

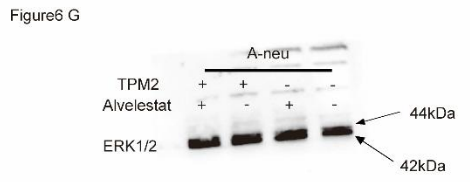

Original Figure 6G- p-ERK1_2

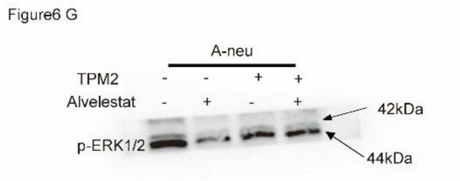

Original Figure 6I ERK1/2:

Figure 6I

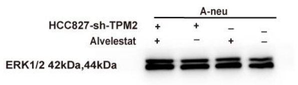

Original Figure 6I p-ERK1/2:

Figure 6I

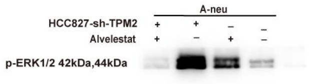

Original Figure 6I GAPDH:

Figure 6I

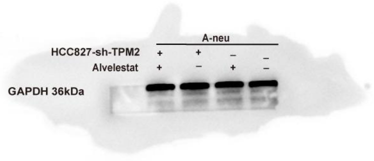

Original Supplementary Figure 2

Original Supplementary Figure 2 PR3-1:

Supplementary Figure 2

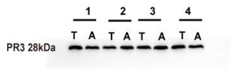

Original Supplementary Figure 2 PR3-2:

Supplementary Figure 2

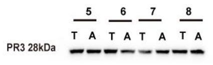

Original Supplementary Figure 2 PR3-3:

Supplementary Figure 2

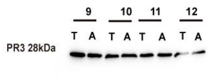

Original Supplementary Figure 2 CTSG-1:

Supplementary Figure 2

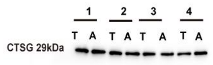

Original Supplementary Figure 2 CTSG-2:

Supplementary Figure 2

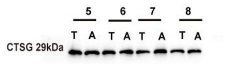

Original Supplementary Figure 2 CTSG-3:

Supplementary Figure 2

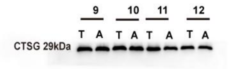

Original Supplementary Figure 2 Granzyme A-1:

Supplementary Figure 2

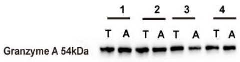

Original Supplementary Figure 2 Granzyme A-2:

Supplementary Figure 2

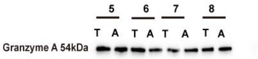

Original Supplementary Figure 2 Granzyme A-3:

Supplementary Figure 2

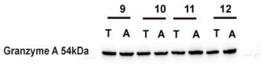

Original Supplementary Figure 2 Granzyme GAPDH-1:

Supplementary Figure 2

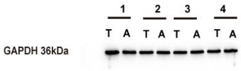

Original Supplementary Figure 2 Granzyme GAPDH-2:

Supplementary Figure 2

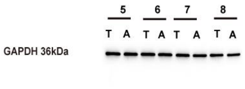

Original Supplementary Figure 2 Granzyme GAPDH-3:

Supplementary Figure 2

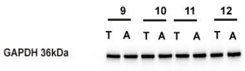

Original Supplementary Figure 3

Original Supplementary Figure 3A TPM2:

Supplementary Figure 3A

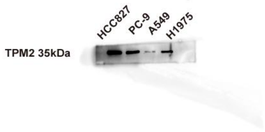

Original Supplementary Figure 3A GAPDH:

Supplementary Figure 3A

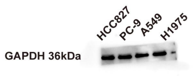

Original Supplementary Figure 3B TPM2:

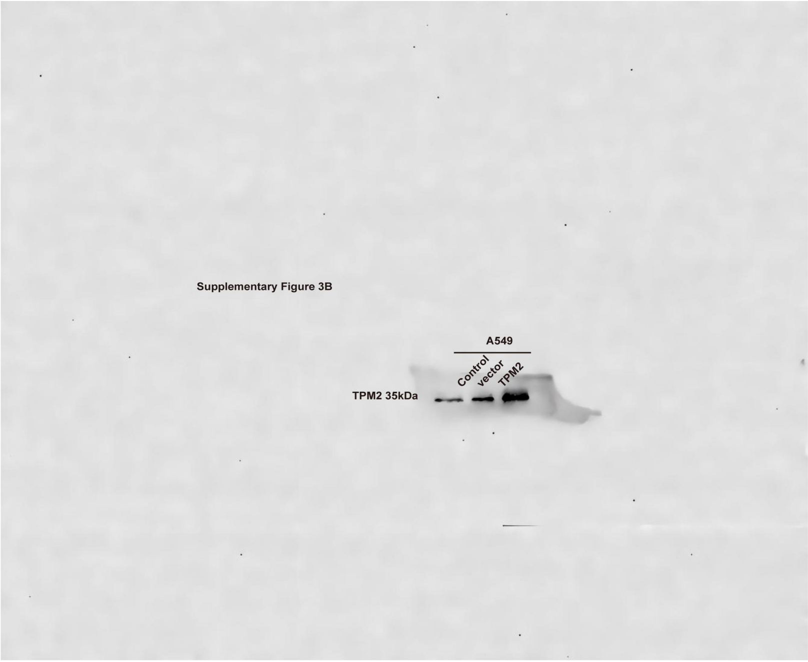

Original Supplementary Figure 3B GAPDH:

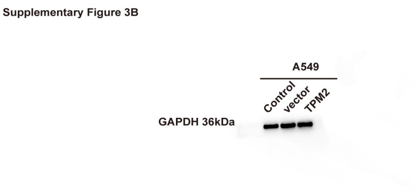

Original Supplementary Figure 3C TPM2:

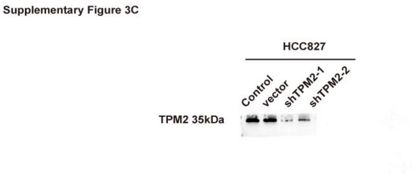

Original Supplementary Figure 3C GAPDH:

Supplementary Figure 3C

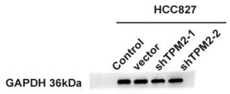

Supplement: Supplementary file 1 — Original western blots [file 41419_2025_7531_MOESM1_ESM.pdf]
