# Supplementary material for: Downregulation of tropomyosin 2 promotes the progression of lung adenocarcinoma by regulating neutrophil infiltration through neutrophil elastase
Source: Cell Death Dis. 2025 Apr 8;16(1):264. doi: 10.1038/s41419-025-07531-1 (PMC11978998; doi:10.1038/s41419-025-07531-1)
Supplement: Supplementary file 2 — Supplementary Figure legend [file 41419_2025_7531_MOESM2_ESM.pdf]

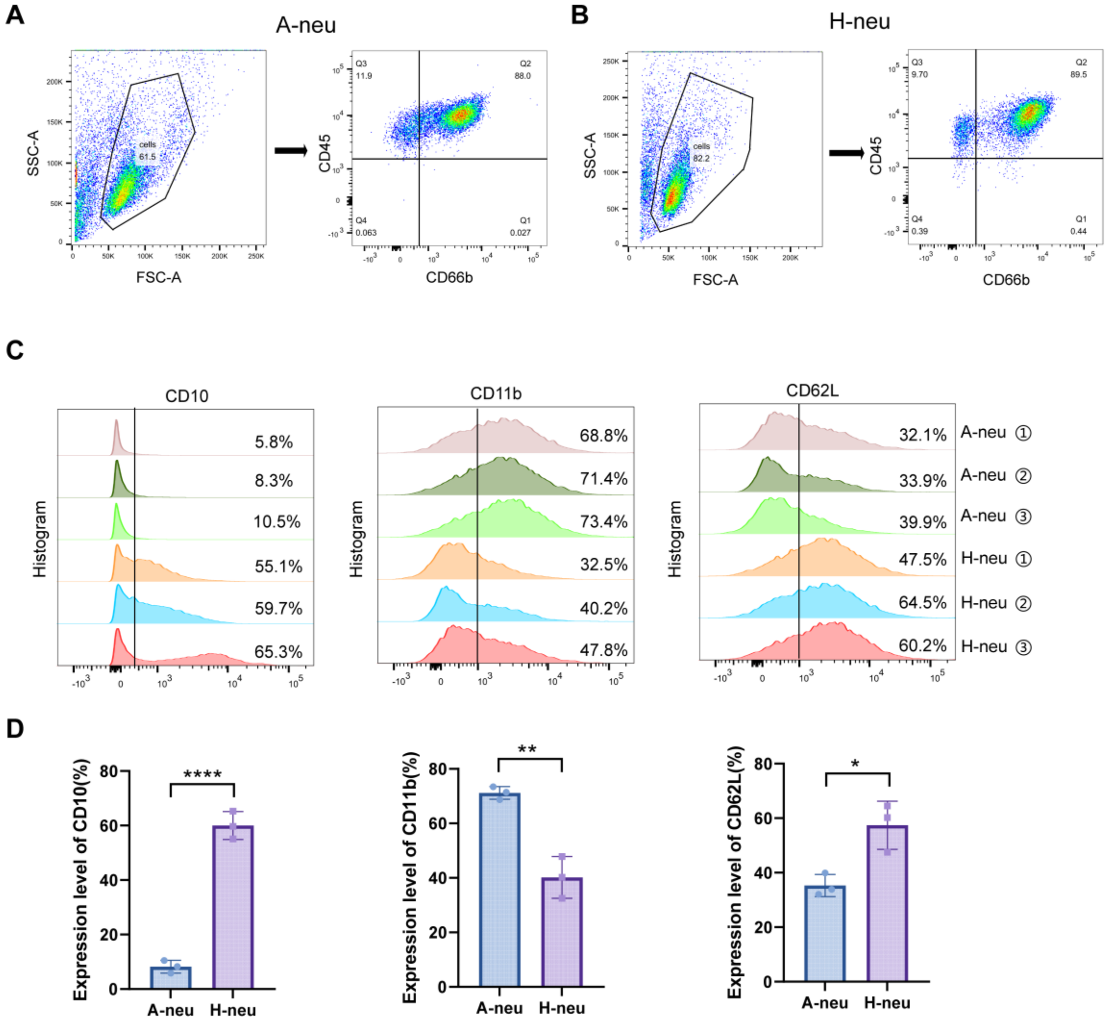

Supplementary Figure 1. LUAD patients with distant metastasis had lower expression of CD10 and CD62L and higher expression of CD11b on neutrophils in peripheral blood.

A, B. Peripheral blood neutrophils from isolated healthy donors and LUAD patients with distant metastases were detected by flow cytometry.

C, D. The expression of CD10, CD11b and CD62L on neutrophils in peripheral blood of three healthy donors and LUAD patients with distant metastasis was detected by flow cytometry.

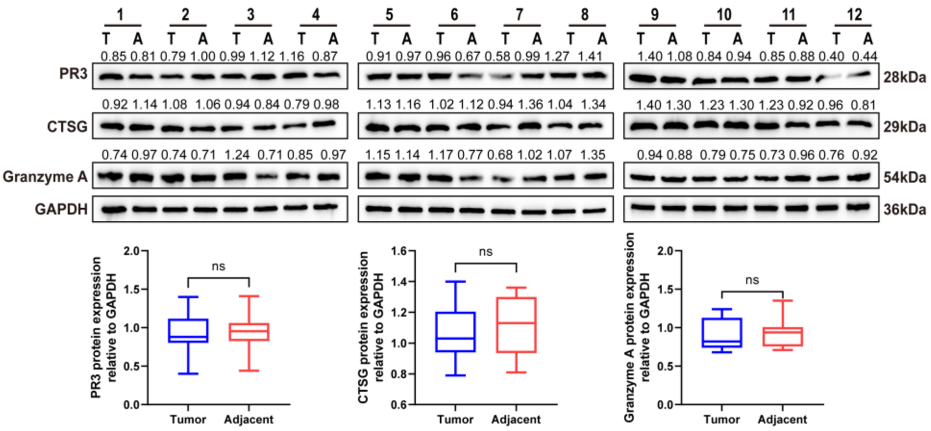

Supplementary Figure 2. The expressions of CTSG, PR3 and granzyme A protein in 12 pairs of LUAD tissues and their adjacent tissues were analyzed by Western blot.

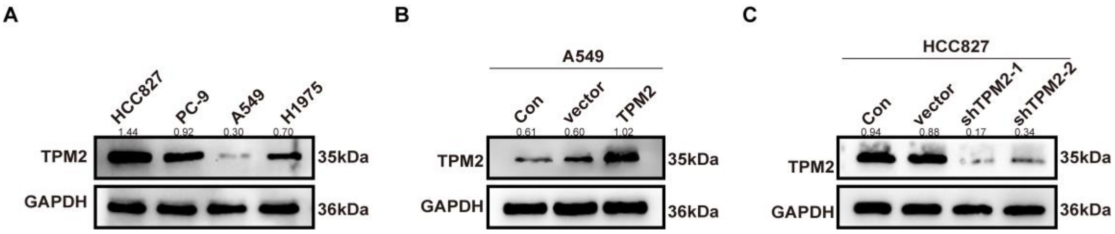

Supplementary Figure 3. Downregulation of TPM2 enhance tumor progression by acting on p38-MAPK pathway to promote ELANE expression in neutrophils, Related to Fig. 5.

A. The protein levels of TPM2 were determined in 4 LUAD cell lines through western blotting.

B, C. The generation of stable cell lines in which TPM2 was overexpressed or silenced was confirmed using western blotting.

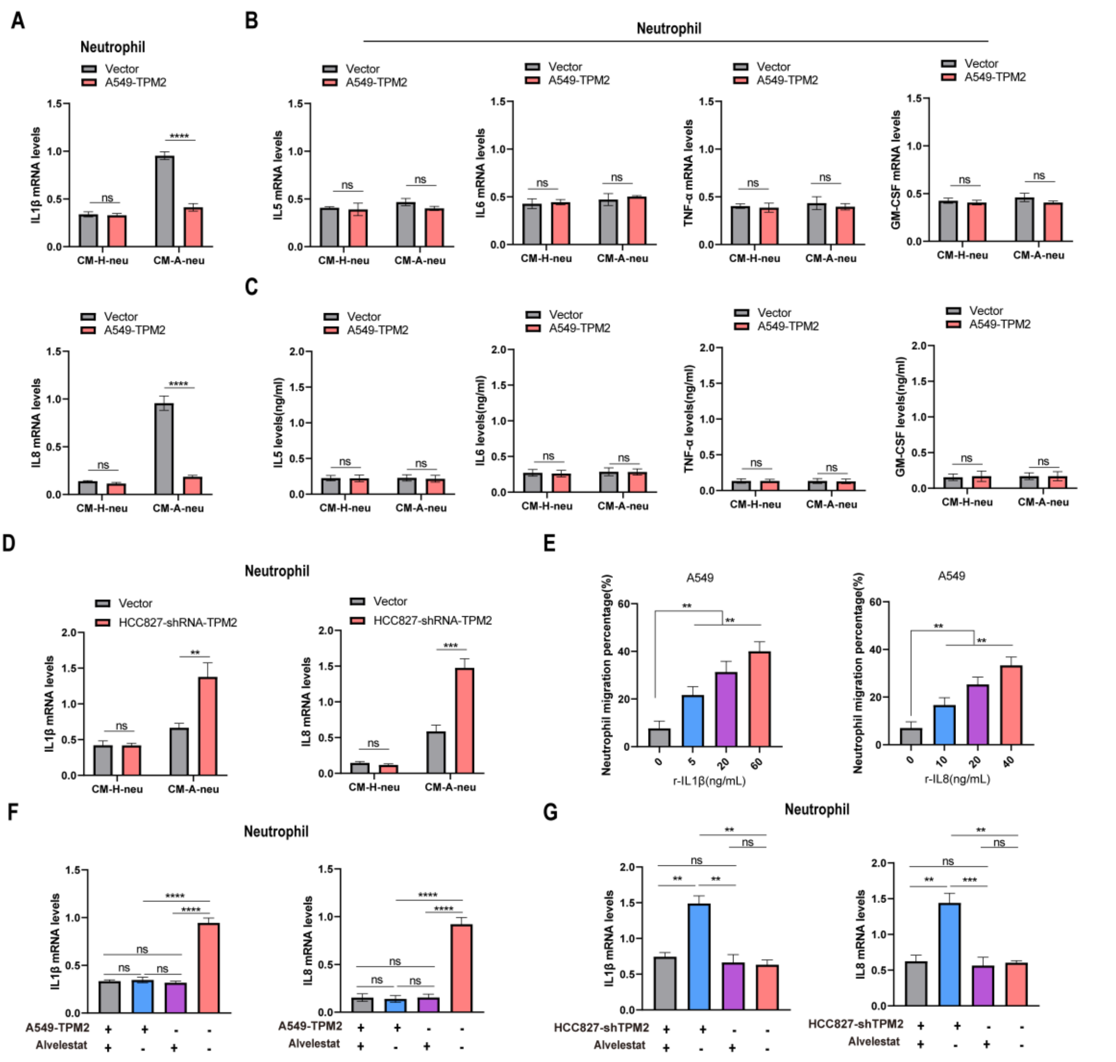

Supplementary Figure 4. Downregulation of TPM2 promotes neutrophil recruitment to tumor sites by activating the ELANE-ERK1/2-IL1β/IL8 axis of neutrophils, Related to Fig. 6.

A. mRNA levels of IL1β, IL8 in A-neu and H-neu after pretreated with vector or TPM2-overexpressed A549 cells.

B, C. mRNA and protein levels of IL6, IL5, GM-CSF and TNFα in A-neu and H-neu after pretreated with vector or overexpressed A549 cells.

D. mRNA levels of IL1β and IL8 in A-neu and H-neu after pretreated with vector or TPM2-konckdown HCC827 cells.

E. Migration of Peripheral blood-derived neutrophils recruited by conditioned medium of neutrophils obtained from A-neu pretreated with A549 combined with different

concentrations of recombinant human IL1β and IL8.

F, G. mRNA levels of IL1β and IL8 in A-neu after pretreated with TPM2-overexpressed A549 cells or TPM2-konckdown HCC827 cells combined with 20 μmol/l ELANE inhibitor (Alvelestat).
