# Supplementary figures and images for: Downregulation of tropomyosin 2 promotes the progression of lung adenocarcinoma by regulating neutrophil infiltration through neutrophil elastase
Source: Cell Death Dis. 2025 Apr 8;16(1):264. doi: 10.1038/s41419-025-07531-1 (PMC11978998; doi:10.1038/s41419-025-07531-1)

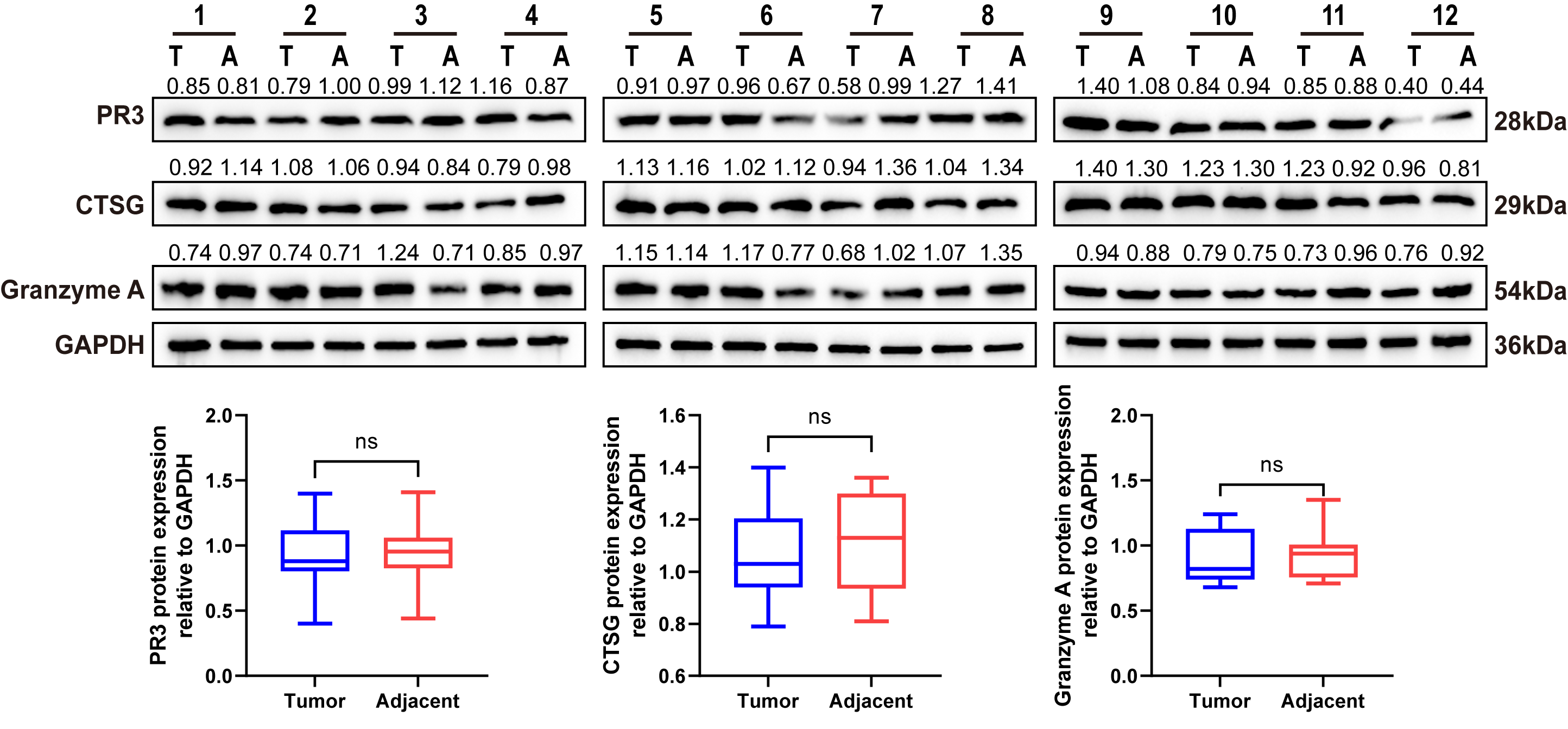

Supplement: Supplementary file 4 — Supplementary Figure 2 [file 41419_2025_7531_MOESM4_ESM.tif]

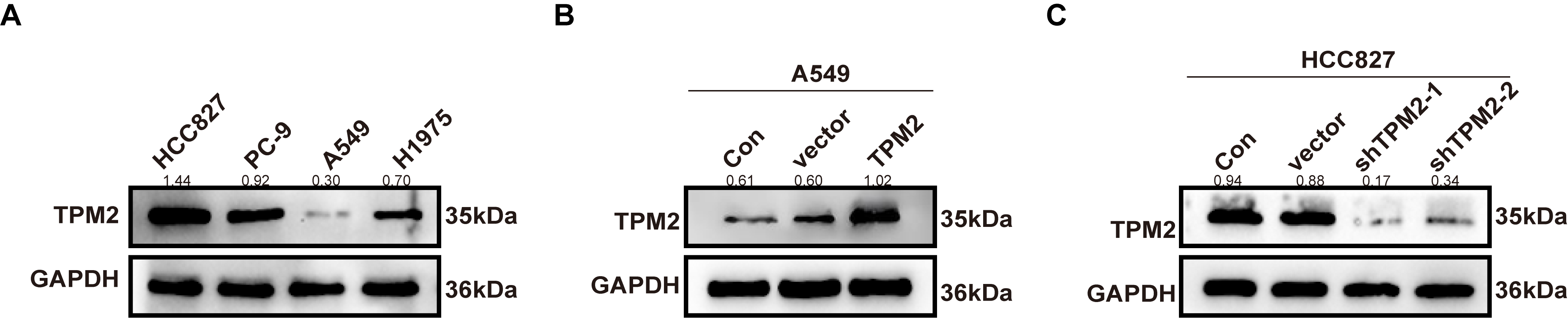

Supplement: Supplementary file 5 — Supplementary Figure 3 [file 41419_2025_7531_MOESM5_ESM.tif]

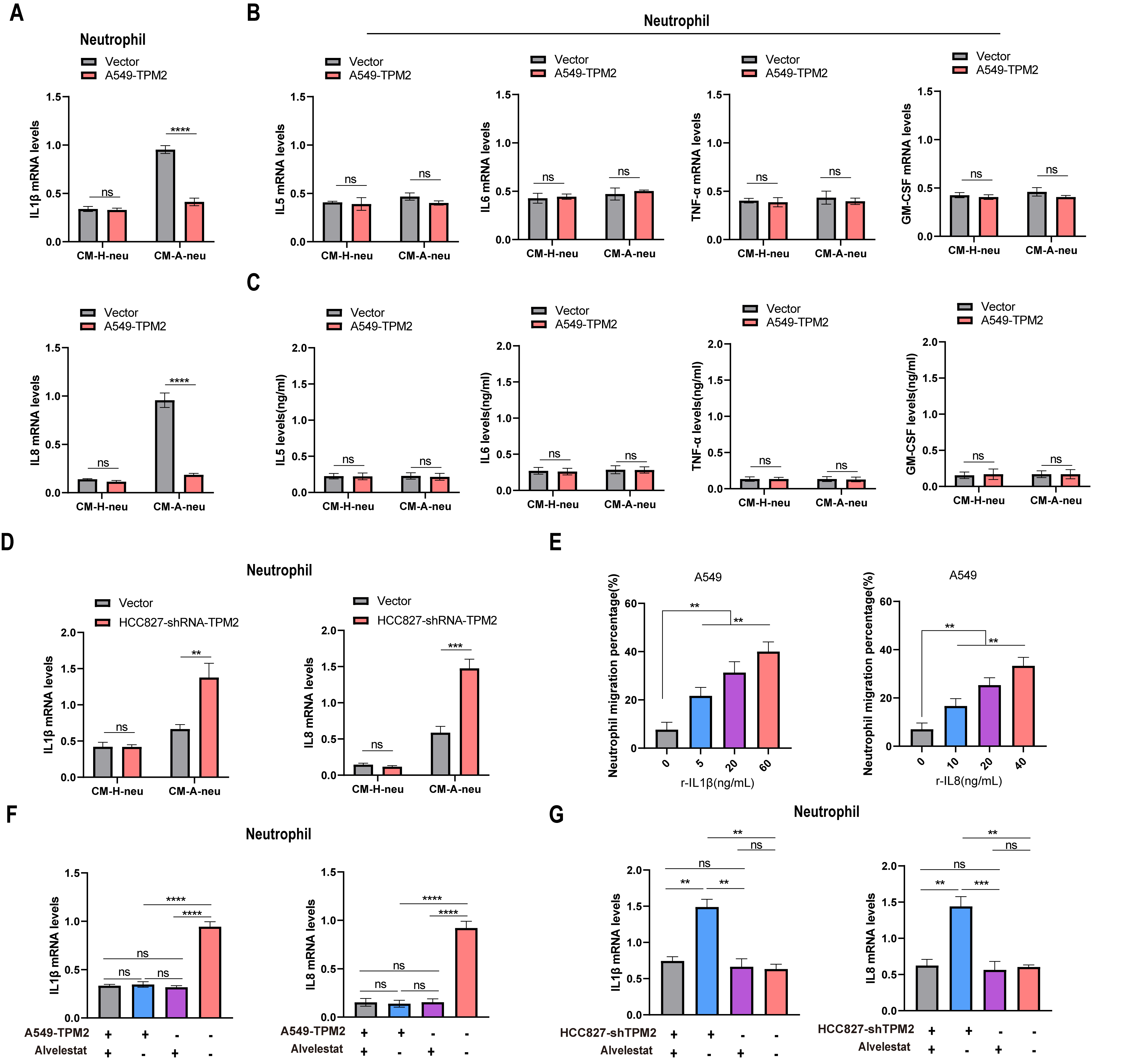

Supplement: Supplementary file 6 — Supplementary Figure 4 [file 41419_2025_7531_MOESM6_ESM.tif]
